# Long-Term Effects of Mountain Hiking vs. Forest Therapy on Physical and Mental Health of Couples: A Randomized Controlled Trial

**DOI:** 10.3390/ijerph20021469

**Published:** 2023-01-13

**Authors:** Daniela Huber, Johanna Freidl, Christina Pichler, Michael Bischof, Martin Kiem, Renate Weisböck-Erdheim, Gabriella Squarra, Vincenzo De Nigris, Stefan Resnyak, Marcel Neberich, Susanna Bordin, René Zechner, Arnulf Hartl

**Affiliations:** 1 Institute of Ecomedicine, Paracelsus Medical University Salzburg, 5020 Salzburg, Austria; 2 Certified Nature and Forest Therapy Guide, 39010 Tisens, Italy; 3 Certified Forest-Health-Trainer, 83435 Bad Reichenhall, Germany; 4 Institute of Sports Medicine, South Tyrol Health Authority, 39100 Bozen, Italy

**Keywords:** nature-based therapy, health-promoting interventions, green exercise, mountain hiking, forest therapy, sedentary lifestyle, climate therapy, health-related quality of life (HRQOL), quality of relationship, psychological and physiological parameters, COVID-19 pandemic, ANKER-study

## Abstract

**Highlights:**

What are the main findings?

- Highly functioning adults with a sedentary lifestyle benefit physically and mentally from forest therapy and mountain hiking.
- Women predominantly benefited highly from mountain hiking regarding hemopoietic system and aerobic capacity. Both genders profited mentally from contact with nature.

What is the implication of the main finding?

- Forest therapy and mountain hiking could be safe and health-promoting interventions for high-functioning adults with a sedentary lifestyle and could be applied in primary prevention as well as in secondary prevention.

**Abstract:**

Background: Lifelong physical activity is related to longer health span, which is reflected at an individual level, and is of substantial socioeconomic relevance. Sedentary lifestyles, on the other hand, pose an increasingly major public health problem. In addition, the COVID-19 pandemic had a negative impact on activity levels and well-being. Previous research indicates that contact with nature might improve exercise levels as well as well-being. Methods: This randomized, controlled clinical trial (ANKER-study) investigated the effects of two types of nature-based therapies (forest therapy and mountain hiking) in couples (FTG: n = 23; HG: n = 22;) with a sedentary or inactive lifestyle on health-related quality of life, relationship quality and other psychological and physiological parameters. Results: The results of this study displayed that healthy and highly functioning women and men with sedentary lifestyles mentally benefit from contact with nature (quality of life, satisfaction with life, mood, internal and external health-related control beliefs). The gender-specific effect on women is most visible in the physiological outcomes (hemopoietic system, aerobic capacity, skeletal muscle mass and hydration) of mountain hiking. Men and women showed small improvements in blood pressure as a result of the interventions. Conclusions: The ANKER-study provides a method for valid comparison of forest therapy interventions for the first time. Regarding the COVID-19 pandemic, the nature-based intervention presented could offer a multimodal contribution to maintaining a more active lifestyle, further contact with nature that affects peoples physical as well as mental health, and an improvement in social interaction.

## 1. Introduction

There is a considerable body of evidence on sustaining health benefits of being active and a scientific consensus that physical activity—in adequate dosage—should be used in the promotion, maintenance and recovering of physical as well as mental health [1,2,3]. Lifelong physical activity is associated with a longer health span and can delay, respectively, prevent the beginning of chronic diseases [4], which is not only relevant on an individual level, but also of significant socio-economic relevance [5].

In order to promote the enjoyment of physical activity and, thus, encourage adherence and support a healthy lifespan for all individuals, it is important to consider different lifestyles [1]. Particular focus should be placed on sedentary or inactive lifestyle, whereas sedentary behavior is described as waking behavior spent in a sitting or lying position with energy expenditure of ≤1.5 METs, and inactive behavior describes an insufficient extent of moderate-to-vigorous physical activity [6]. These lifestyles pose a major public health problem, with almost a third of the world’s population now behaving in an inactive way [7], whereby, underlying factors are multifaceted [8]. The effects of such passive behavior are also manifold, e.g., time spent sitting is a major risk factor for mortality and morbidity [9]. For example, physical inactivity, together with low energy consumption and overeating, are seen as causes of obesity, which is also associated with unemployment, social disadvantage, and reduced socio-economic productivity and a growing economic burden since the last 50 years [10]. Recognizing the strong link between physical activity and the major noncommunicable diseases and their impact on global health, WHO Member States have agreed on a 10 % relative reduction in the prevalence of insufficient physical activity by 2025 to improve the prevention and treatment of noncommunicable diseases [11]. Nevertheless, if these current trends of inactive lifestyles continue, this mark will not be realized [12].

Clinically relevant health benefits for a wide range of conditions could be achieved at levels of physical activity clearly below current international recommendations (e.g., least 150 min of weekly moderate to vigorous physical activity) [7]. However, achieving the minimum suggested level is reported to be associated with near-maximum longevity benefits too [2]. Already, for adolescent populations, there is a significant association between increased levels of physical activity and improved self-rated health [13]. In addition, findings showed that the COVID-19 pandemic had a negative impact on activity levels, sleep quality, and well-being in a group of physically active people [14], with changes in work behavior with teleworking in inactive positions also contributing here [15].

Therefore, effective lifestyle interventions should take an integrated approach to health and promote the importance of healthy lifestyles such as reducing sedentary time and increasing physical activity, additionally promoting healthy eating as well as managing stress [1]. Furthermore, improving social relationships should be integrated in such a multimodal concept, as human social connections to others have a powerful impact on health and longevity; a lack of social connections on the other hand is associated with a risk for premature mortality [16]_._

Further, evidence shows that exposure to immunoregulatory microorganisms (“Old Friends”) is essential for humans to decrease immune (re)activity and inflammation [17,18]. Following the “biodiversity hypothesis”, reduced human contact with natural environmental features and biodiversity may negatively impact the human commensal microbiota and its immunomodulatory capacity [19,20]. Insufficient contact with immune-regulating microorganisms and biodiversity with which humans have co-evolved may result from an urban environment with little access to natural resources [17,21]. This is supposed to be especially the case in high urbanized countries with high incomes—and consequently high hygiene standards, since in low-income countries, where people are still exposed to a high degree to microbes from the environment, the immunoregulatory effect of “Old Friends” is more pronounced [22]. Although, the evidence regarding the positive influence of environmental microbial exposures on human health is encouraging, further research is needed to assess the potential impact of “Old Friends,” including environmental microbial influences, on biological characteristics and clinically meaningful prevention and intervention outcomes for health [23].

Additionally, predominantly in high-income countries, a significant decline in physical activity has been observed (insufficient activity in 2001: 31.6%, in 2016: 36.8%). Globally, more than a quarter of the population has insufficient levels of physical activity. Furthermore, beyond reduced contact with nature, urbanization thus leads to lifestyle changes as work and leisure activities shift indoors, encouraging inactive or sedentary behavior [12]. These complex problems of inactivity and lack of access to immune-regulating microorganisms and biodiversity need to be given special attention in terms of disease prevention and treatment, as urbanization is rapidly increasing worldwide: while only about one third of the world’s population lived in urban settlements in 1970, this is expected to rise to over 60 % by 2030 [24].

A possible approach to reconnect with nature and “Old Friends” as well as to increase physical activity, could be Green Exercise. This means exercise in nature, as it is the case in particular with mountain hiking or, in a less intensive form, with forest therapy. Mountain hiking can be described as a long-lasting activity of moderate intensity at different altitudes [25]. This leisure activity has become very popular [25]: every year, millions of people of all ages decide to spend their holidays hiking in Alpine regions [26,27]. Accordingly, natural zones, including national parks and nature reserves, are becoming areas of interests in recreational activities and tourists increasingly [28]. The natural environment itself has an important role in mountain hiking, because proximity to nature is a key factor for practicing mountain hiking [29]. However, the ideal characteristics of a natural environment for a targeted health-promoting use are still unclear. Even anthropogenic elements such as ski lifts, fences or buildings seem to have little influence on acute stress-related physiological responses and affective states during mountain hiking [30]. Additionally, the altitude itself has a positive influence during hiking: an active vacation at moderate altitude (1500–2500 m) has lasting positive effects on adult health; it improves the quality of sleep, well-being and physical recovery [31].

Likewise, the positive effect of nature and its specific elements on human health is the essential component of forest therapy: This approach of nature connection therapy, also known as “forest bathing”/”Shinrin-Yoku”, is a collective term for activities designed to improve human health or well-being in a forest environment. The crucial element of forest therapy is recognition for the forest environment using the five human senses, which can be combined with meditation, breathing exercises, forest walks, various recreational activities, and cognitive behavioral therapy [32]. Additionally, results from Forest Therapies showed that in the natural environment, participants first experienced positive emotional changes, followed by cognitive and behavioral changes, which may also be relevant in terms of moving away from a sedentary or passive lifestyle [33]. 

Forest therapy has been shown to be effective in improving immune function; a hypothesis for this is that the forest biodiversity and microbiome has a significant impact; more precisely, phytoncides from trees can reverse stress-induced immunosuppression and thus, normalize immune function and neuroendocrine hormone levels [34], which may also be of interest with respect to the COVID-19 pandemic: This hypothesis can be complete, as—for example—proportionally fewer COVID-19 deaths in the 2020 and 2021 observation period in southern Italy and on the Italian islands are also explained by forest bathing, Mediterranean environment, and antiviral volatile organic plant compounds [35].

Although, there are further studies on positive health effects of forests [36,37,38], more research is still needed, both, conceptually and methodologically [39], where a clinically relevant patient benefit should be prioritized [40]. 

The purpose of the ANKER-study (“Algund Nature and Climate Therapy: Green Exercise vs. Nature Connection”) is to investigate long-term effects of two types of nature-based therapies—mountain hiking vs. forest therapy—in couples with a sedentary lifestyle on health-related quality of life (HRQOL), relationship quality and other psychological and physiological parameters.

## 2. Materials and Methods 

### 2.1. Study Design and Settings

The present data were collected as part of the ANKER-Study (ISRCTN43292449); this randomized, controlled clinical trial investigated the effects of two types of nature-based therapies in couples with a sedentary lifestyle on health-related quality of life, quality of relationship, psychological and physiological parameters. Therefore, two intervention groups—a hiking group (HG) and a forest therapy group (FTG)—were included. The allocation ratio was set at equal sample size. The study protocol was approved by the Ethics Committee of Bolzano, Italy (reference number 18-2019) and can be accessed with further details on materials and methods at https://doi.org/10.3390/ijerph19073888, accessed on 1 October 2022 [40]. 

### 2.2. Participants

Couples with a sedentary lifestyle were the defined group of interest in the ANKER-Study, therefore, participants had to meet the following inclusion criteria: age 50–60 years, relationship-duration > 1 year, BMI ≥ 25 and ≤30, sedentary lifestyle (International Physical Activity Questionnaire Short Form (IPAQ-SF) < 3.00 METmin/week) and the ability to participate in moderate hiking tours (Physical Activity Readiness Questionnaire/PAR-Q). The following exclusion criteria were applied: active lifestyle, immunologically mediated chronic conditions or immunodeficiency, severe respiratory diseases, acute or untreated psychiatric disorders, uncontrolled hypertension, uncontrolled metabolic disease, acute infection or fever, diagnosis of or treatment for malignant neoplastic disorders within the last 5 years, arteriosclerotic event < 6 months before enrollment, cardiac insufficiency, renal insufficiency, diagnosis or history of alcoholism, current recreational drug use, currently smoking > 10 cigarettes/day, orthopedic contraindications for hiking, medication intake > 5mg/d prednisone, colchicine, Imuran, methotrexate, azathioprine, cyclophosphamide or cyclosporine, intake of weight-loss drugs or preparations as well as pregnancy. By selecting people between 50 and 60 years of age, the interventions studied could contribute to maintaining the ability to work in this age group, as this would be a primary preventive measure to maintain health. The participating couples were recruited online via webpage (https://www.klimatherapie.eu/, accessed on 1 October 2022) and advertisement on social media channels between April 2019 and May 2021 allover Austria and Germany. Due to limited human resources, the recruitment and, also, the intervention periods had to be extended as described, as scientific staff performed the extensive measurements on site. The seven-day vacation with mountain hiking and forest therapy in South Tyrol/Italy itself was the incentive to participate.

### 2.3. Intervention

Participants of both intervention groups spent a seven-day vacation in Algund (Italy, 46°40′57.5″ N 11°07′19.0″ E, 350 m AMSL), a region that is characterized by its mild, nearly Mediterranean climate. All participants were housed in local hotels, got the same meals, and did not receive lifestyle recommendations during the non-intervention phase.

The HG took part in guided, moderate hiking tours every day (3–4 h), except for one rest day in the middle of the intervention week. The nature group received daily 3–4 h of standardized nature connection therapy sessions with a psychologist trained for this purpose. Forest therapy was characterized by low physical activity and oriented on a holistic framework that promoted meaningful connections at the three levels of “connection with nature”, “connection with others”, and “connection with oneself”. In forest therapy, each day had a specific theme that was worked on (mindfulness and relaxation, connection to nature, social connections, connection to self, goal setting and next steps). After a practice phase, the participants finished the intervention each day with a written self-reflection. These interventions were conducted in two separate, but identical sequences: The first part of the study population completed the ANKER-Study in October 2019, the second part in June 2021.

### 2.4. Data Collection and Outcomes

**Figure 1:**
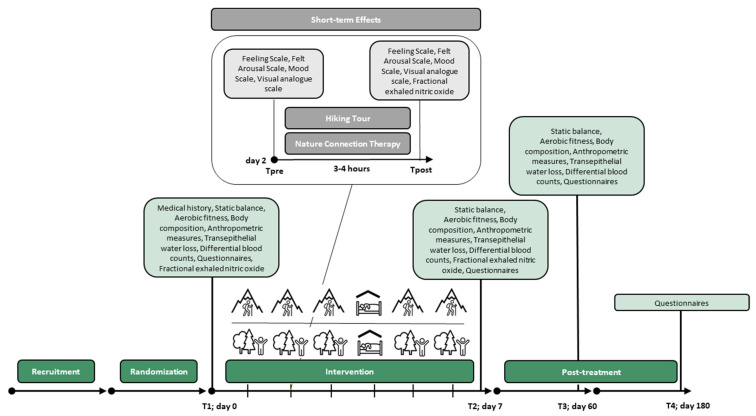
Study schedule [41,42].

Medical examinations at T1 (day 0; before the intervention), and T2 (day 7; after the intervention) were performed at the Department of Sports Medicine, Tappeiner Hospital, Merano (Italy). Follow-up examinations at T3 (day 60) took place at the Paracelsus Medical University, Salzburg (Austria); the follow-up examination at T4 (day 180) was carried out as an online survey. More details are shown in Figure 1.

The ANKER study investigated the following long-term effects on T1, T2, T3 and T4: Health-related quality of life (Short form health survey (SF12), EuroQol (EQ-5D-5L), Quality of relationship (Partnership Questionnaire (PFB) and Problem List (PL)) as primary outcomes. A further primary outcome—the Intercultural Quality of Life Comic will become part of a separate paper due to its extensive results and an additional validation (throughout the following questionnaires: Subjective impairments of the participants (Complaints list (B-L’), BFI-10 questionnaire (10 Item Big Five Inventory) and Health-related control beliefs (HLOC) in this context. 

Secondary parameters were nature connectedness (Connectedness to Nature Scale (CNS), Nature Relatedness Scale (NRS) and Inclusion to Nature Scale (INS)), sociopsychological well-being in the sense of flourishing of personality (Flourishing Scale (FS-D), life satisfaction (Satisfaction with Life Scale (SWLS), health-related control beliefs (FEGK), subjective impairments of the participants Mindfulness (Mindful Attention and Awareness Scale (MAAS). Measurements of the International Physical Activity Questionnaire Short Form (IPAQ-SF) were collected at T1, T3 and T4 to assess a possible long-term impact of the intervention on the subjects’ physical activity behavior. Additionally, the following physiological parameters were collected on T1, T2 and T3: static balance (MFT-S3 Check), body composition via Bio Impedance Analysis (Four-terminal impedance analyzer), differential blood count (Forearm venous blood), fractional exhaled nitric oxide (FeNO; NioxMino^®^), aerobic fitness (Chester step test) and transepithelial water loss (TEPWL, Tewameter^®^ TM 300). Additionally, anthropometric measures (height, weight, waist and hip circumference) were acquired. The motion profile of the heart rate monitors (watches on the wrists of the subjects) was obtained over the time of the intervention. 

The short-term effects collected as part of the ANKER-study will be presented in a follow-up paper due to the quantity and consequent complexity of these data.

### 2.5. Statistical Analysis

All statistical analyses were performed in the sense of an intention-to-treat analysis using the R-GNU software environment (General Public License, R Foundation for Statistical Computing, Vienna, Austria). Statistical significance was set at the level of α < 0.05. Baseline data were analyzed using unpaired Students-*t*-Test or Wilcoxon-test, depending on normal distribution.

Fully nonparametric variance type tests (ANOVA type statistics) from the nparLD (Nonparametric Longitudinal Data Analysis) package [43] were used to evaluate long term effects. To identify possible gender effects, F2-LD-F1 model with group and gender as whole-plot factor and time as subplot factor (T1-T4) were defined. In case of a significant gender effect, the female and male subgroup were analyzed separately by F1-LD-F1 models with group, time and group*time. Otherwise, females and males were analyzed together. Within the F1-LD-F1 model, group was defined as whole plot factor and time as sublot factor. In case of a significant time effect, another F1-LD-F1 model was applied as post hoc test. Relative treatment effects were used as a measure of effect: a RTE > 0.5 in the HG indicates a tendency for participants of this group to score at least as high or higher as a randomly chosen subject from the total population. On the other hand, a RTE of 0.25 in the HG means that the probability of a randomly chosen participant from the total population having a lower score than a randomly chosen person from the exercise group is estimated to be 25%.

### 2.6. Randomization and Sample Size

Pairwise randomization of the couples to groups was completed according to the following stratification factors: Age, general health (PHQ-9), closeness to nature (NRS-6), BMI, activity level (IPAQ-SF) and relationship duration [44]. Due to the nature of the intervention, no blinding was conducted. The sample size was estimated to be 39 persons per group using health-related quality of life data from a previous intervention study (ISRCTN18092043) [45] (ANOVA with fixed effects, main effects and interaction: effect size f = 0.38, type I error α = 0.05, power 1−β = 0.85, number of groups = 2, degree of freedom = 2).

## 3. Results

### 3.1. Study Participants and Baseline Characteristics

Two hundred and fifty-seven individuals were screened for eligibility to participate in this study, 165 were excluded: 49 individuals did not meet the inclusion criteria, 116 individuals declined to participate due to personal reasons or the COVID-19 pandemic. 

**Figure 2:**
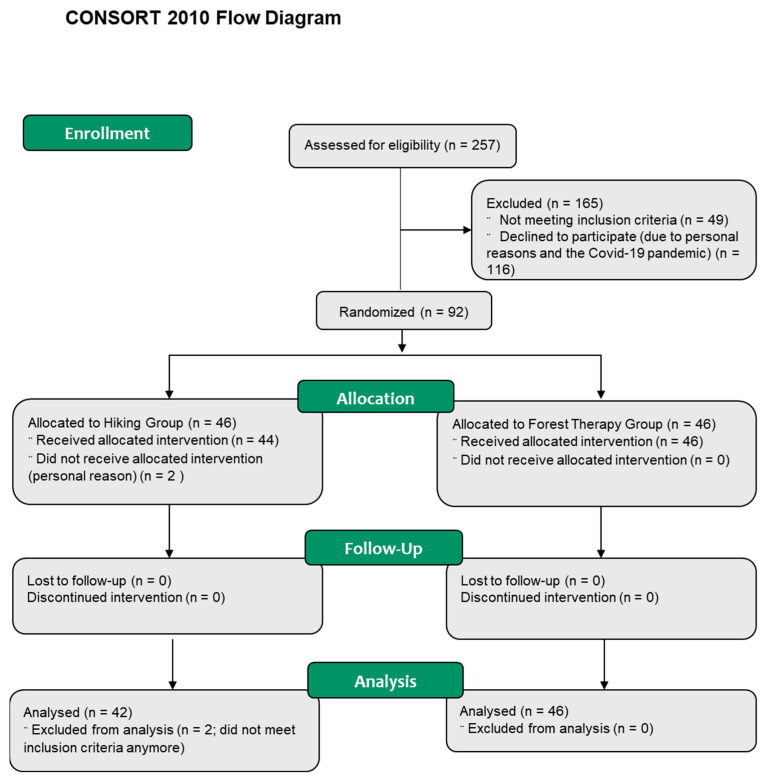
Study flowchart of included and excluded patients.

In October 2019, 24 people were enrolled in the HG and 28 in the FTG; in June 2021, 20 people were enrolled in the HG and 18 in the FTG. This time gap occurred due to limited scientific staff resources. The four groups were pooled into one HG (n = 42) and one FTG (n = 46) for examination. Two participants from the HG did not receive allocated intervention (personal reasons) and two further participants were excluded from the analysis because they did not meet inclusion criteria anymore (relationship status). Consequently, 42 participants of the HG and 46 participants of the forest therapy group (Figure 2) were included for the intention-to-treat-analysis.

Except for relationship duration, baseline characteristics showed no relevant differences in inclusion criteria between the study groups (Table 1). In the FTG, relationship duration was slightly longer than in the HG (Mann–Whitney U test, *p* = 0.06, W = 742). An extended overview of all other baseline characteristics is summarized in Tables S1 and S2 in the supplementary materials.

The hiking intervention as well as the forest therapy were well tolerated by all participants. No adverse effects or harms were observed in any group. 

### 3.2. Tour Data

On average, the mountain hikes lasted 03:29 h, the participants covered a distance of 7.36 km and 521 m of altitude. The FTG participated each day in standardized forest therapy sessions for 3–4 h, assisted by a psychologist. The sessions were characterized by low physical activity (Table 2). Both interventions could be carried out as planned.

### 3.3. Primary Outcomes

Results from the F1-LD-F1 analysis of primary outcomes are accessible in Table 3. The F2-LD-F1 model revealed no gender-specific effects for any primary outcome. Both groups presented with high baseline levels for health-related quality of life and quality of relationship. 

The F1-LD-F1 model for the SF-12 total score revealed a significant main effect for time (*p* < 0.01). Post hoc tests did not show any interaction effects at the single time points, indicating a parallel improvement in both groups. The SF-12 Physical Component was rated significantly higher by the HG at baseline than by the FTG (*p* = 0.02, W = 1240.5). Significant main effects for group (*p* < 0.01) and time (*p* < 0.01) were observed. Post hoc test did not show any interaction effects at the single time points. The parallel RTE-profile in combination with the significant baseline difference indicates again a parallel improvement in both groups. For the SF-12 Mental Component, a significant main effect for time was found (*p* = 0.02), indicating a parallel development in both groups. 

The F1-LD-F1 model for the EQ5D-5L visual analogue scale revealed a significant main effect for time (*p* < 0.01). Post hoc test showed no interaction effects at the single time points, thus indicating a parallel improvement in both groups. The EQ5D-5L Index presented with a significant main effect for group (*p* = 0.02) and time (*p* < 0.01). A post hoc test failed to reveal any interaction effects at the single time points, thus indicating together with the RTE profile a parallel improvement in both groups until day 60. The improvement continues in the HG, whereas the EQ-5D-5L index decreases in the FTG towards day 180.

Regarding the quality of relationship (PFB total score), both groups start with high baseline levels (HG: 61.10 ± 12.24, FTG: 59.64 ± 13.13). Scores ≥ 54 are considered as a mostly happy relationship. The F1-LD-F1 model for the PFB Total Score revealed a significant main effect for time (*p* < 0.01). Post hoc test revealed no interaction effects at the single time points. The relatively flat RTE profiles indicate, that the quality of relationship did not change in any group throughout the study. The F1-LD-F1 model for the subscale happiness revealed a significant main effect for time (*p* < 0.01) and a trend for group*time (*p* = 0.07). Post hoc test failed to identify any interaction effects. Again, the relatively flat RTE-profile indicates, that the happiness subscale did not change throughout the study. A significant main effect for time (*p* = 0.02) and for group*time (*p* = 0.03) was identified for the problem list (PL). No interaction effects at the single time points were found. However, the participants in both groups rated at all four time points only 1–2 relationship areas as ≥ 2.

### 3.4. Differential Blood Count

Table 4 shows the results from the F1-LD-F1 analysis for the values of differential blood count, vital parameters and skin quality. The F2-LD-F1 model identified gender-specific effects for erythrocytes (*p* < 0.01) and hematocrit (*p* < 0.01). 

The F1-LD-F1 model for erythrocytes identified a significant main effect for time (*p* < 0.01) and group*time (*p* < 0.01) in the female subgroup. Post hoc test indicates a significant decrease in erythrocytes in the female HG towards day 7 (*p* = 0.04) with a return to baseline at day 60 (*p* = 0.08). Within the male subgroup, only a significant main effect for time (*p* < 0.01) was found. Post hoc test did not show any interaction effects. However, the RTE-profile also indicates a decrease in erythrocytes in the male HG.

Within the female subgroup, a significant main effect for time (*p* < 0.01) and group*time (*p* = 0.03) was identified for hematocrit. The male subgroup presents with a significant main effect for group (*p* = 0.02) and time (*p* < 0.01). Post hoc test and RTE profile indicate a decrease in hematocrit for both sexes in the HG towards day 7 with a return to baseline at day 60.

In contrast, the F1-LD-F1 model for reticulocytes showed an opposite dynamic. A main effect for time (*p* < 0.01) indicated an increase towards day 7 and a return to baseline at day 60 in both groups. However, the immature reticulocyte fraction (IRF) presented with a significant main effect for time (*p* < 0.01) and group*time (*p* = 0.05). Post hoc test and RTE profile indicated an increase in the HG towards day 7 (*p* = 0.07) and a return to baseline at day 60.

Statistical analysis of the white blood cell count revealed a significant main effect for time (*p* < 0.01), indicating anti-inflammatory effects of both interventions. Post hoc test did not show any interaction effects at the single time points. 

### 3.5. Aerobic Capacity, Balance, Vital Parameters and Skin Quality

Table 4 displays the results from the F1-LD-F1 analysis for aerobic capacity. Although no gender-specific effects were identified for aerobic capacity by F2-LD-F1 model, females and males were analyzed separately as aerobic capacity depends on gender.

**Figure 3:**
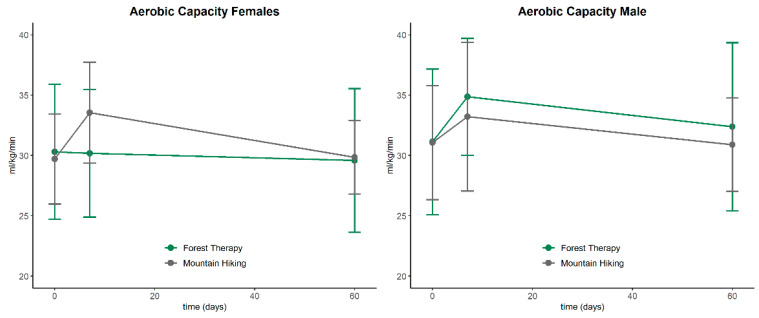
Mean ± standard deviation of aerobic capacity for the female and male subgroups on days 0, 7 and 60 (forest therapy vs. mountain hiking).

F1-LD-F1 model for aerobic capacity in the female subgroup revealed a significant main effect for time (*p* = 0.04) and group*time (*p* = 0.03). Post hoc test found a significant interaction effect at day 7 (*p* < 0.01) indicating an improvement in the HG. Within the male subgroup, a significant main effect for time (*p* = 0.04) was found. Post hoc test failed to show any interaction effects at the single time points. However, the RTE profile indicates an improvement in aerobic capacity in the male FTG. a graphical representation, see Figure 3.

Participants in both groups already reach around 77% in the S3-Check Stability index at the beginning of the study. A significant main effect for time (*p* < 0.01) indicates an improvement in both groups. However, the improvement is in the HG (T2-T1: 1.16%) and FTG (T2-T1: 2.63%) rather small. In contrast, the sensorimotor index did not show any significant changes. 

F1-LD-F1 models for blood pressure found similar effects for systolic and diastolic blood pressure. A significant main effect for time (*p* < 0.01) indicates a decrease in systolic blood pressure in both groups (HG T1-T2: 7.78 mmHg, FTG T1-T2: 6.15 mmHg). Diastolic blood pressure presented also with a significant main effect for time (*p* = 0.02), indicating an improvement (HG T1-T2: 3.75 mmHg, FTG: T1-T2: 1.98 mmHg). F1-LD-F1 models for pulse and SpO_2_ revealed significant main effects for time (*p* < 0.01), but post hoc tests failed to show interaction effects at the single time points. No significant group-, time- or interaction effects for skin parameters (skin hydration and transepithelial water loss) were observed. In Table S3, the results of the F1-LD-F1 analysis of balance, vital parameters and skin quality can be found.

### 3.6. Body Composition

Results from the F1-LD-F1 analysis of Body Parameters are presented in Table S4. F2-LD-F1 models revealed significant gender-specific effects for resistance (*p* < 0.01), reactance (*p* < 0.01), phase angle (*p* < 0.01), weight (*p* < 0.01), fat mass index (*p* < 0.01), fat free mass index (*p* < 0.01), skeletal muscle mass index (*p* < 0.01), body cell mass index (*p* < 0.01) and hydration (*p* < 0.01).

F1-LD-F1 model for body mass index revealed a significant main effect for time (*p* < 0.01) and group*time (*p* = 0.05). Post hoc test did not show any interaction effects. Although a significant time effect was found, the changes in BMI are rather small and lack any clinical relevance. Additionally, body weight presents in both sexes with a significant main effect for time (female *p* = 0.04, male *p* < 0.01) but lacking any clinical relevance. 

F1-LD-F1 model for reactance revealed a significant main effect for time (*p* < 0.01) and group*time (*p* = 0.01) in the female subgroup. The male subgroup presented only with a main effect for time (*p* < 0.01). F1-LD-F1 models for resistance found for both subgroups a significant main effect for time (female *p* = 0.01, male *p* = 0.05). No significant effects were found for phase angle in any group. 

Within the female subgroup, the Fat Free Mass Index presented with a significant main effect for time (*p* < 0.01) and group*time (*p* = 0.03), indicating an increase in fat free mass in the HG. Furthermore, the Skeletal Muscles Index revealed also a significant main effect for time (*p* < 0.01) and group*time (*p* = 0.01) in the female subgroup, indicating an increase in skeletal muscle mass in the HG. The F1-LD-F1 model for hydration presented with a significant main effect for time (*p* = 0.02), indicating an increase in hydration in the HG. No effects were found for fat mass index.

Within the male subgroup, significant main effects for time were found for Fat Free Mass Index (*p* < 0.01) and Skeletal Muscles Index (*p* < 0.01). No effects were found for hydration and fat mass index. 

### 3.7. Questionnaires

F2-LD-F1 models revealed gender-specific effects for the connectedness to nature scale (CNS, *p* = 0.01) and flourishing scale (FS-D, *p* = 0.04). For all these questionnaires, it should be noted that the participants already had high scores initially and consequently, the changes in the scores were minor.

Nature connectedness increased in both intervention groups as significant main effects for time were found for the CNS, INS and NRS-6. F1-LD-F1 model for MAAS Total revealed only a significant main effect for time (*p* < 0.01), indicating a parallel improvement in both groups. Internal health-related control beliefs (FEGK internal) revealed a significant main effect for group (*p* = 0.01) and time (*p* = 0.02). Post hoc test did not find any interaction effects at the single time points. External health-related control beliefs (FEGK external) presented with a main effect for time (*p* = 0.01). Within the female subgroup, the flourishing scale revealed a significant main effect for time (*p* = 0.01) indicating together with the RTE-profile a personal flourishing in the HG. No significant effects were found for the male subgroup. F1-LD-F1 models for the IPAQ-SF revealed significant main effects for time, indicating a parallel development in both groups. The physical activity increased in both groups slightly throughout the study. For further details, all questionnaire data can be found in Table S5.

### 3.8. Sample Size Simulation

**Figure 4:**
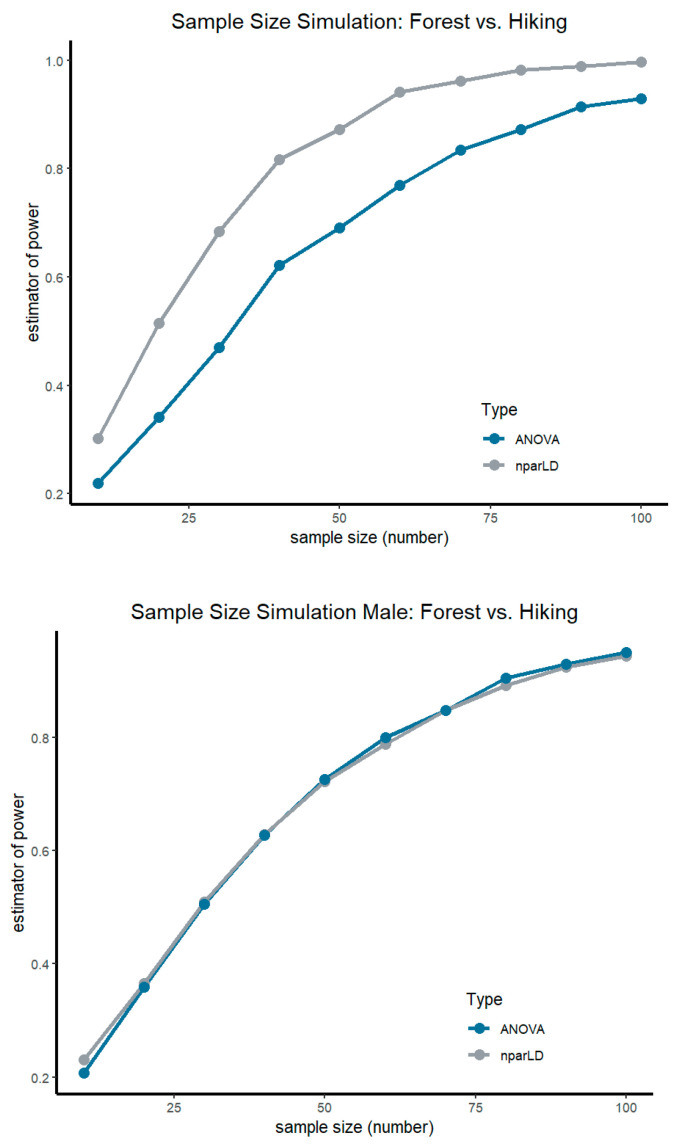

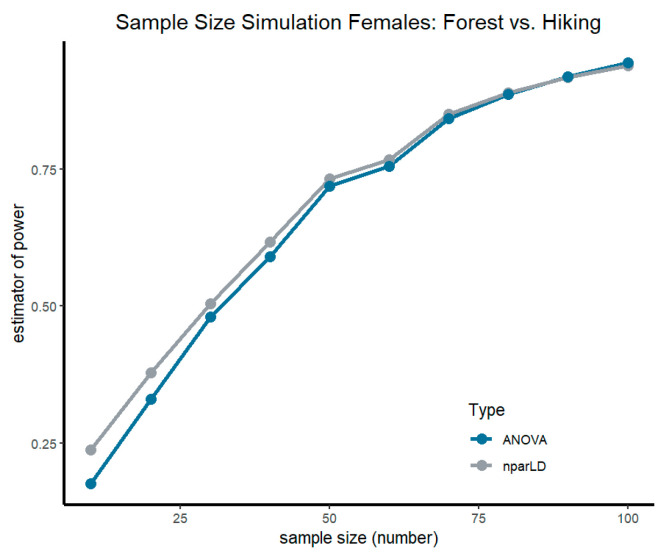
Sample size simulation based on data of the ANKER-Study. Upper graphic shows the comparison of the two intervention groups (forest therapy vs. mountain hiking) for primary outcome SF12 Total Score. The graphs below present gender-specific analyses of aerobic capacity for woman and men. Sample size simulation was performed for nparLD and ANOVA.

Consistent with the bootstrap sample size simulation for the primary outcome SF12 Total Score, a sample size of at least n = 50 per group is needed to achieve a power of 1−β = 0.87 in the case nparLD analysis for the comparison of the two interventions, mountain hiking and forest therapy. A larger sample size is required for analysis using ANOVA−at least n = 80 per group is required to achieve a power of 1−β = 0.87 (see Figure 4). The gender-specific comparison between the Mountain HG and the FTG in terms of aerobic capacity reveals the following results: Females require at least an n of 70 (1−β = 0.85) in a nparLD analysis, whereas males require an n of at least 80 (1−β = 0.89). With respect to an ANOVA analysis, women (1−β = 0.89), similarly to men (1−β = 0.91), need an n of at least 80.

## 4. Discussion

The ANKER-Study was conducted before and during the COVID-19 pandemic and investigated the effects of two different types of nature-based therapies in couples with a sedentary lifestyle. Effects of mountain hiking and forest therapy on health-related quality of life, relationship quality, and other psychological and physiological parameters were investigated. The COVID-19 theme was taken up in this paper, as this pandemic influenced not only organizational aspects, but it also changed the perspective towards gender-specific implications, as the impact of this pandemic is esteemed meaningfully higher for women than for men. Women, especially working women, are disproportionately responsible for most domestic tasks, including care for children and elderly [46,47].

Current research on COVID-19 and human-nature relationships, indicates that women are more likely to attribute higher values to nature and recreation than men [46]. This different rating could be explained by gender roles—women attribute higher personal relevance to mental wellbeing and communicate mental health issues more openly [46,48]. In contrast, our results showed that both sexes benefit mentally from contact with nature in terms of quality of life (SF-12, EQ5D-5L), mood, satisfaction with life as well as internal and external health-related control beliefs. Gender-specific results were only found for the flourishing scale (FS-D). Personal flourishing improves predominately in the female HG. No changes were found for the male subgroup. However, no significant differences were found between the HG and FTG for any of these parameters, indicating that being outdoors in natural environments is the driving force behind the improvements of mental indicators. The subjects’ relationship quality proved to be a stable construct, improving only slightly after the intervention, but without clinical relevance. However, it should be noted here that there is a ceiling effect among the cohort. Given, that the COVID-19 pandemic can also have an impact on the quality of couple’s romantic relationships [49], these interventions presented in the ANKER-study can be considered a stabilizing element. It would be of research interest how this could further improve relationship quality, particularly of couples with previously low relationship quality.

The results with regard to physiological parameters, on the other hand, were different regarding gender: Since both interventions took place in altitudes of ~1500 m, their hematopoietic system was stimulated; as this exposed the subjects to the interactions between low barometric pressure and partial-pressure of O_2_ to an incipient adaptation and acclimatization processes of altitude [50]. However, this effect was even stronger in the HG. Here, the activation of the hemopoietic system became especially clear in women of the HG. In combination with the increase in reticulocytes, it became apparent how mountain hiking led to an improved exchange of old red blood cells. Additionally, when fat-free mass was considered separately in men and women, a differentiated picture emerged: Again, the HG benefited more from the intervention, women again more significantly than men. This was also reflected in the increase in aerobic capacity, skeletal muscle mass and hydration, mainly in the HG. Thus, it can be assumed that women predominantly benefit physiologically more from this kind of green exercise intervention.

Vital signs were kept as physiological control parameters in this study, whereby a very positive trend was observed for blood pressure in both gender groups: In our study, persons with sedentary lifestyle initially showed a slightly elevated blood pressure (HG: 135.40 ± 19.07/83.38 ± 10.20 bpm, FTG:132.30 ± 16.80/82.83 ± 10.66 bpm), which already approached the recommended range immediately after the one-week intervention (HG: 130.74 ± 13.36/81.90 ± 8.01bpm, FTG:129.80 ± 16.27/81.50 ± 9.63bpm), so a clinically relevant change can be assumed. Gatterer et al. stated that in elderly persons with hypertension, moderate intensity hiking only at weekends may reduce systolic blood pressure; otherwise, it did not improve cardiovascular risk factors in healthy older people. They did not find gender-specific differences [51]. Similarly, Neumayer et al. found significant reductions in systolic, diastolic and mean arterial pressure in male patients with metabolic syndrome, as well as circadian heart rate profiles, after a three-week holiday program with 12 hikes at moderate altitude (at 1700 m) or low altitude (200 m) [52]. In contrast, it was observed by Stoltzfus et al. that during prolonged exposure to moderate altitude (1980–3960 m), there is an increase in adult participants’ mean blood pressure during a 10-day hiking intervention, especially in women. Therefore, to increase the safety of mountaineers from cardiac events, mountain hiking has been recommended for individuals with a modestly controlled blood pressure until 160/95 mmHg [53]. In our subjects, the blood pressure range was controlled and maintained to ensure the safety of the subjects. Due to the moderate altitude, it can be assumed by the results that hiking, as it was performed in this study, is a safe intervention for the selected population. Additionally, forest therapy is particularly interesting in this context: In cases, safe mountain hiking is not advisable due to internal or coordinative conditions or restricted mobility (altitude, physical resilience, risk of falling), this nature-based intervention also has the potential to improve health and well-being. What is more, our results showed that stability was increased in both intervention groups in men and women, which in turn may be an additional benefit for people with poor mobility. Nevertheless, the safety of individuals and protection from injury must always be paramount.

Forest therapy in the ANKER-study was professionally guided by a specialized psychologist. Findings exhibited that guided forest therapy programs promote positive emotional changes and social bonding through interaction with others [54]. Which, in terms of a multimodal intervention, may be beneficial for individuals with sedentary lifestyles to maintain or move toward a more active lifestyle. Furthermore, this is also highly relevant with regard to COVID-19 because social isolation can have a significant contribution to compromising people’s health and well-being [55,56]. Especially these nature-based therapies, which are performed outdoors with distance, can also be a safe alternative to indoor group activities to promote social interaction as well. On the other hand, self-guided forest therapy is attributed to provide a better opportunity for self-reflection. In this regard, a variation of forest therapy would be recommended, with intermittent inputs by professional guidance and periods of self-guided activity in between, in order to obtain the best realizable effects, and to support an active lifestyle that lasts as long as possible [54]. A combination of guided exercises and “solo exercises” was also repeatedly applied in the FTG of the present study: In these sessions, the guide gave a very short input at the beginning, afterwards participants had the possibility to get into self-experience (e.g., “Medicine Walk”, “Sit-spot”, “Communication with a tree”, etc.) for a period of time (about 20–40 min).

Moreover, there are also differences in the gender-specific influence of COVID-19: Amplified risk for developing mental health problems for women during the pandemic is experienced with pregnancy or intimate partner violence [47]. In contrast, during the confinement in the COVID-19 pandemic it affected mainly very active men as well as young people and students that the daily physical activity decreased, and sedentary time increased [57]. Moreover, several studies have highlighted the gender differences in prevalence and comorbidity profiles between men and women infected with COVID-19. In summary, men demonstrated a higher prevalence of pulmonary and cardiovascular disorders, while women had a higher prevalence of obesity and renal conditions [58]. In addition to biological factors, gender differences in behavior and lifestyle may also contribute [59]. Roviello and Roviello [35] generated the hypothesis that forest therapy may also be prophylactic against COVID-19: Their modeling showed that plant organic compounds, such as those found in a forest, could bind and disturb the complex that the receptor binding domain of the coronavirus spike protein forms with the human cell receptor. Based on this reasoning, forest therapy and also mountain hiking could also be preventive to improve physical and mental health, which in turn could have also an impact on the comorbidity and consequently mortality of COVID-19 infections. This could be the subject of further research.

During the COVID-19 pandemic, concerned women, older persons, college-educated respondents, and white-collar workers noted the greatest use of the forest as a recreation site. Nonetheless, women and people with elementary education expressed the most fears related to visiting the forest. In contrast, men and people who lived in rural areas and small towns, as well as respondents who performed activities related to the forest, were most involved in exploring and working in the forest and [60]. Other findings also suggest that nature can be an important resource for people in times of crisis, but that the specific interactions and associated values that individuals feel are the most important may vary among population groups [46]. Evidence shows that forest therapy programs can promote the health of middle-aged women, prevent disease, and improve quality of life. Health is especially critical for women of this age, who are exposed to more stress than at other stages of life, thereby affecting quality of life in old age [61]. In postmenopausal women, on the other hand, forest therapy can be a good alternative to non-pharmacological treatment to alleviate related insomnia [62]. Additionally, even in young women, a walk in a forest could lead to physiological and psychological relaxation effects [63]. These results indicate that women particularly benefit from the health-promoting effects of forest therapy or conscious stays in forests. In this regard, it is also important to consider gender-specific interventions that both genders may perceive the forest and nature as a health resource in daily living as well as during a crisis.

### Strengths and Limitations

To enhance the methodological quality and evidence, while decreasing the risk of bias, as required in reviews on the topic of forest therapy [36,38,64], this study was not only well-designed and planned according to the SPIRIT statement [65], but additionally, enabled a comparability of interventions and results for further studies: Thus, a survey form for the forest profile was created to make forest therapy classifiable regarding characteristics of the forest and thus more comparable (https://doi.org/10.3390/ijerph19073888 access on 1 October 2022). In this context, it would be of interest to know what influence the forest characteristics themselves have on the results, here further research and comparisons with other forests are needed. The ANKER-study was conducted in a small forest area, with mixed, medium old and rather high stock of trees in a quiet environment [40].

Nature relatedness in the ANKER-Study was assessed via three questionnaires (Connectedness to Nature Scale, Inclusion to Nature Scale and Nature Relatedness Scale). The results indicated that connectedness to nature is a stable personality characteristic, as the scores changed only slightly, even though significance occurred over time, i.e., low clinical significance can be expected. Furthermore, it could be assumed that there was a selection bias in the present study population, because especially persons with a high affinity to nature would volunteer to participate in such a study design, which is also reflected in the high, stable values of these three questionnaires. 

As the bootstrap sample size simulation based on the results of the ANKER study shows, a sample size of n ≥ 80 persons is recommended in order to similarly match the psychological and physiological parameters of nature-based interventions. Although, in this study only about half of the recommended subjects participated, significant results could be obtained already, therefore higher case numbers are likely to generate even clearer and clinically more relevant results. Additionally, further research specific to women with regard to mountain hiking and also forest therapy should be conducted to further elucidate the effects of these interventions on female gender and to ascertain their backgrounds, e.g., fears related to visiting the forest [60]. Furthermore, to verify the validity of the results presented, a control group would be needed to exclude other confounding factors, this is recommended for further studies.

Buckley and Brough [66] pointed out that there is ample evidence that nature experiences and activities can prevent, delay, or ameliorate the mental health components of chronic illnesses. Nevertheless, to make nature therapies more practical and, also importantly, prescribable, dose–duration–effect relationships are lacking. With the ANKER-study, long-term effects of forest therapy and mountain hiking for sedentary, healthy and highly functioning persons can be offered now. Equally, this is also valid for the physiological parameters of this study. Consequently, further research on individuals with pre-existing mental or physiological health conditions regarding these interventions is recommended, again for a working-adult population, in order to consider the possible socio-economic effects accordingly. Specific diagnostic tests, questionnaires and observations need to be developed within this framework to adapt and prescribe these two types of Nature Connection Therapies for individual patients as well [67].

## 5. Conclusions

The ANKER-study, with its forest classification survey, provides for the first time a method for a more valid comparison of forest therapy interventions. The long-term results of this study showed that healthy, highly functioning women and men with sedentary lifestyles benefit mentally from contact with nature. The gender-specific effect of mountain hiking on women is most visible in physiological parameters. Further research should investigate forest therapy as a health-promoting intervention for individuals with immobility or internal diseases. Advanced gender-specific research, especially with regard to these interventions on women, is also recommended. Regarding the COVID-19 pandemic, the nature-based intervention presented could offer a multimodal contribution to maintaining or creating a more active lifestyle, further contact with nature that affects people’s physical as well as mental health, and an improvement in social interaction. Overall, focus should be on high quality research to make these types of nature-based therapies more applicable and prescribable for a working-adult population.

## Figures and Tables

**Table 1:** Baseline characteristics of the study population regarding inclusion criteria.

	Hiking Group (n = 42)		Forest Therapy Group (n = 46)		Baseline Tests	
	Mean ± SD	Median ± IQR	Mean ± SD	Median ± IQR		
Gender	male n = 21	female n = 21	male n = 23	female n = 23	1	χ² Test
Age (years)	58.57 ± 5.14	59 ± 7	58.89 ± 5.67	60 ± 9	0.67	U-Test
Duration of relationship (years)	22.57 ± 13.84	26 ± 26	28.39 ± 11.66	33 ± 18.25	0.06	U-Test
BMI (kg/m^2^)	27.46 ± 2.04	27.61 ± 2.88	27.90 ± 3.48	27.09 ± 3.68	0.86	U-Test
IPAQ-Short Score (MET-min/week)	1757.78 ± 1768.94	1074.25 ± 1845.5	4853.12 ± 14,078.25	974 ± 3609.13	0.82	U-Test
NRS-6	21 ± 5.6	21.5 ± 8	21.83 ± 4	22 ± 4	0.52	U-Test
PHQ-9	2.71 ± 2.56	2 ± 3	3.41 ± 3.17	3 ± 4	0.31	U-Test

BMI: Body Mass Index, IPAQ-Short: Score of International Physical Activity Questionnaire in metabolic equivalent (MET) minutes per week, NRS-6: Nature Relatedness Scale 6, PHQ-9: Patient Health Questionnaire.

**Table 2:** Characteristics of the intervention program.

		Mountain Hiking				Forest Therapy *		
Time	Intervention	Duration (hours/min)	Distance (km)	Altitude ** (hm)		Duration (hours/min)	Distance (km)	Altitude (m)
Day 1	1	04:07	12.6	*↑334*	*↓334*	03:10	-	-
Day 2	2	03:06	8.0	*↑214*	*↓787*	03:50	-	-
Day 3	3	04:01	10.0	*↑639*	*↓639*	03:50	-	-
Day 4		*Recovery*				*Recovery*		
Day 5	4	03:38	9.8	*↑569*	*↓569*	03:45	-	-
Day 6	5	02:30	4.8	*↑745*	*↓745*	03:20	-	-
*Average*		*03:28*	*9.04*	*↑500*	*↓615*	*03:35*	*Max. 3*	*Max. 100*

* The values in the FTG can only be given as an average—they vary depending on the participant and individual execution of the exercises. ** The difference in ascent and descent was caused by the use of a mountain cable car.

**Table 3:** Results from the F1-LD-F1-model for primary outcomes.

Parameter	F1-LD-F1 Model				Relative Treatment Effects (RTE)						Descriptive Statistics	
		F	*p*	adj. *p*	Time		Hiking		Forest Therapy		Hiking	Forest Therapy
SF-12	Group	2.89 (1.00, ∞)	0.09				Hiking	0.55	Forest T.	0.46	mean ± SD	mean ± SD
Total Score	Time	8.07 (2.61, ∞)	<0.01 **		T1	0.43	Hi. x T1	0.47	FT. x T1	0.40	82.90 ± 9.32	81.17 ± 8.05
	Group*Time	0.45 (2.61, ∞)	0.69		T2	0.53	Hi. x T2	0.57	FT. x T2	0.49	85.31 ± 10.54	83.95 ± 8.51
	Group*T2	0.04 (1.00, ∞)	0.84	0.84	T3	0.54	Hi. x T3	0.60	FT. x T3	0.48	86.88 ± 8.07	83.49 ± 8.33
	Group*T3	1.07 (1.00, ∞)	0.30	0.60	T4	0.51	Hi. x T4	0.56	FT. x T4	0.46	85.56 ± 9.46	82.24 ± 9.91
	Group*T4	0.43 (1.00, ∞)	0.51	0.60								
SF-12	Group	8.22 (1.00, ∞)	<0.01 **				Hiking	0.57	Forest T.	0.43	mean ± SD	mean ± SD
Physical	Time	12.48 (2.76, ∞)	<0 01 **		T1	0.41	Hi. x T1	0.48	FT. x T1	0.34	85.89 ± 8.80	81.30 ± 9.51
Component	Group*Time	0.78 (2.76, ∞)	0.05		T2	0.52	Hi. x T2	0.58	FT. x T2	0.46	88.57 ± 9.13	85.22 ± 9.31
	Group*T2	0.36 (1.00, ∞)	0.55	1.00	T3	0.55	Hi. x T3	0.61	FT. x T3	0.48	89.52 ± 8.47	85.22 ± 10.70
	Group*T3	0.02 (1.00, ∞)	0.89	1.00	T4	0.54	Hi. x T4	0.63	FT. x T4	0.44	90.36 ± 7.52	84.13 ± 10.66
	Group*T4	0.91 (1.00, ∞)	0.34	1.00								
SF-12	Group	0.39 (1.00, ∞)	0.53				Hiking	0.52	Forest T.	0.48	mean ± SD	mean ± SD
Mental	Time	3.31 (2.66, ∞)	0.02 *		T1	0.46	Hi. x T1	0.46	FT. x T1	0.46	80.69 ± 11.29	81.08 ± 10.37
Component	Group*Time	0.69 (2.66, ∞)	0.54		T2	0.53	Hi. x T2	0.54	FT. x T2	0.51	82.89 ± 13.34	83.01 ± 10.52
	Group*T2	0.73 (1.00, ∞)	0.39	0.78	T3	0.53	Hi. x T3	0.57	FT. x T3	0.49	84.92 ± 9.62	82.21 ± 10.14
	Group*T3	2.24 (1.00, ∞)	0.13	0.40	T4	0.49	Hi. x T4	0.51	FT. x T4	0.48	82.01 ± 12.56	80.84 ± 12.18
	Group*T4	0.32 (1.00, ∞)	0.40	0.57								
EQ5D-5L	Group	0.40 (1.00, ∞)	0.53				Hiking	0.52	Forest T.	0.49	mean ± SD	mean ± SD
Visual	Time	11.25 (2.80, ∞)	<0.01 **		T1	0.43	Hi. x T1	0.45	FT. x T1	0.40	82.14 ± 11.59	80.87 ± 10.92
Analogue	Group*Time	0.15 (2.80, ∞)	0.92		T2	0.58	Hi. x T2	0.58	FT. x T2	0.57	87.38 ± 10.83	85.22 ± 15.74
Scale (VAS)	Group*T2	0.33 (1.00, ∞)	0.57	1.00	T3	0.52	Hi. x T3	0.54	FT. x T3	0.51	85.95 ± 9.64	83.26 ± 13.34
	Group*T3	0.02 (1.00, ∞)	0.90	1.00	T4	0.48	Hi. x T4	0.50	FT. x T4	0.46	84.05 ± 12.31	81.74 ± 12.88
	Group*T4	0.00 (1.00, ∞)	0.99	1.00								
EQ5D-5L	Group	5.20 (1.00, ∞)	0.02 *				Hiking	0.56	Forest T.	0.45	mean ± SD	mean ± SD
Index	Time	10.19 (2.78, ∞)	<0.01 **		T1	0.44	Hi. x T1	0.48	FT. x T1	0.39	0.90 ± 0.14	0.90 ± 0.07
	Group*Time	0.88 (2.78, ∞)	0.45		T2	0.47	Hi. x T2	0.52	FT. x T2	0.42	0.93 ± 0.07	0.90 ± 0.08
	Group*T2	0.01 (1.00, ∞)	0.92	1.00	T3	0.57	Hi. x T3	0.62	FT. x T3	0.52	0.94 ± 0.07	0.93 ± 0.06
	Group*T3	0.06 (1.00, ∞)	0.81	1.00	T4	0.53	Hi. x T4	0.61	FT. x T4	0.45	0.95 ± 0.06	0.90 ± 0.09
	Group*T4	1.80 (1.00, ∞)	0.18	1.00								
Partnership	Group	0.26 (1.00, ∞)	0.61				Hiking	0.52	Forest T.	0.49	mean ± SD	mean ± SD
Questionnaire	Time	5.71 (2.65, ∞)	<0.01 **		T1	0.50	Hi. x T1	0.51	FT. x T1	0.48	61.10 ± 12.24	59.64 ± 13.13
Total Score	Group*Time	0.15 (2.65, ∞)	0.91		T2	0.53	Hi. x T2	0.54	FT. x T2	0.51	62.64 ± 14.06	60.62 ± 13.62
	Group*T2	0.00 (1.00, ∞)	0.99	1.00	T3	0.52	Hi. x T3	0.53	FT. x T3	0.51	62.31 ± 13.29	60.48 ± 13.51
	Group*T3	0.01 (1.00, ∞)	0.91	1.00	T4	0.46	Hi. x T4	0.48	FT. x T4	0.44	60.02 ± 15.16	57.02 ± 14.91
	Group*T4	0.13 (1.00, ∞)	0.72	1.00								
Partnership	Group	0.52 (1.00, ∞)	0.47				Hiking	0.48	Forest T.	0.52	mean ± SD	mean ± SD
Questionnaire	Time	5.69 (2.55, ∞)	<0.01 **		T1	0.52	Hi. x T1	0.49	FT. x T1	0.55	3.93 ± 0.95	4.14 ± 0.86
Happiness	Group*Time	2.51 (2.55, ∞)	0.07		T2	0.52	Hi. x T2	0.51	FT. x T2	0.53	4.05 ± 0.73	4.03 ± 0.99
	Group*T2	1.17 (1.00, ∞)	0.28	0.84	T3	0.52	Hi. x T3	0.47	FT. x T3	0.57	3.81 ± 1.11	4.20 ± 0.83
	Group*T3	0.63 (1.00, ∞)	0.43	0.85	T4	0.44	Hi. x T4	0.45	FT. x T4	0.42	3.74 ± 1.15	3.67 ± 1.08
	Group*T4	2.33 (1.00, ∞)	0.13	0.85								
Problem List	Group	0.11 (1.00, ∞)	0.74				Hiking	0.49	Forest T.	0.51	mean ± SD	mean ± SD
	Time	3.43 (2.72, ∞)	0.02 *		T1	0.52	Hi. x T1	0.51	FT. x T1	0.53	0.81 ± 1.45	1.04 ± 2.01
	Group*Time	3.12 (2.72, ∞)	0.03 *		T2	0.49	Hi. x T2	0.47	FT. x T2	0.51	0.50 ± 1.02	0.74 ± 1.34
	Group*T2	0.30 (1.00, ∞)	0.58	1.00	T3	0.47	Hi. x T3	0.43	FT. x T3	0.50	0.38 ± 1.08	0.91 ± 2.29
	Group*T3	0.34 (1.00, ∞)	0.34	1.00	T4	0.53	Hi. x T4	0.56	FT. x T4	0.49	1.40 ± 2.47	0.78 ± 1.65
	Group*T4	3.09 (1.00, ∞)	0.08	1.00								

F1-LD-F1 model with group (forest therapy or hiking), time and the interaction of group and time (group*time); T1 = day 0/baseline, T2 = day 7/after intervention week, T3 = day 60/follow-up 1, T4 = day 180/follow-up 2; ** <0.01, * <0.05, n. s./not significant ≥0.05; Results for the Partnership Questionnaire Subscales dispute behavior, tenderness and commonality/communication can be found in Table S5 in the supplemental materials. Abbreviations: adj. *p*: Holm-Bonferroni corrected *p*-value, EQ-5D-5L: Euro Quality of Life Questionnaire (Index variable and Visual analogue scale), F: F-Value, FT.: forest therapy group, Hi.: hiking group, *p*: *p*-value, RTE: Relative Treatment Effects, SD: standard deviation, SF-12: Short Form Health Survey.

**Table 4:** Results from the F1-LD-F1-model for differential blood count and aerobic capacity.

Parameter	F1-LD-F1 Model				Relative Treatment Effects (RTE)						Descriptive Statistics	
		F	*p*	adj. *p*	Time		Hiking		Forest Therapy		Hiking	Forest Therapy
Female	Group	0.26 (1.00, ∞)	0.61				Hiking	0.52	Forest T.	0.48	mean ± SD	mean ± SD
erythrocytes	Time	7.62 (1.83, ∞)	<0.01 **		T1	0.54	Hi. x T1	0.56	FT. x T1	0.52	4.67 ± 0.36	4.61 ± 0.27
(10^6^ µL)	Group*Time	7.37 (1.83, ∞)	<0.01 **		T2	0.44	Hi. x T2	0.40	FT. x T2	0.47	4.48 ± 0.31	4.56 ± 0.35
	Group*T2	5.45 (1.00, ∞)	0.02 *	0.04 *	T3	0.52	Hi. x T3	0.60	FT. x T3	0.45	4.74 ± 0.41	4.52 ± 0.28
	Group*T3	3.07 (1.00, ∞)	0.08	0.08								
Male	Group	1.85 (1.00, ∞)	0.17				Hiking	0.45	Forest T.	0.55	mean ± SD	mean ± SD
erythrocytes	Time	7.50 (1.74, ∞)	<0.01 **		T1	0.56	Hi. x T1	0.52	FT. x T1	0.60	4.88 ± 0.29	4.99 ± 0.40
(10^6^ µL)	Group*Time	1.01 (1.74, ∞)	0.36		T2	0.44	Hi. x T2	0.36	FT. x T2	0.52	4.70 ± 0.28	4.87 ± 0.33
	Group*T2	2.58 (1.00, ∞)	0.11	0.22	T3	0.49	Hi. x T3	0.45	FT. x T3	0.53	4.82 ± 0.28	4.91 ± 0.39
	Group*T3	0.01 (1.00, ∞)	0.92	0.92								
Female	Group	0.24 (1.00, ∞)	0.97				Hiking	0.50	Forest T.	0.50	mean ± SD	mean ± SD
hematocrit	Time	9.92 (1.89, ∞)	<0.01 **		T1	0.52	Hi. x T1	0.53	FT. x T1	0.51	42.01 ± 2.73	41.87 ± 2.08
(g/dL)	Group*Time	3.67 (1.89, ∞)	0.03 *		T2	0.42	Hi. x T2	0.38	FT. x T2	0.47	40.57 ± 2.80	41.46 ± 2.68
	Group*T2	4.03 (1.00, ∞)	0.04 *	0.09	T3	0.56	Hi. x T3	0.60	FT. x T3	0.52	42.74 ± 3.05	41.83 ± 2.19
	Group*T3	0.66 (1.00, ∞)	0.42	0.42								
Male	Group	5.23 (1.00, ∞)	0.02 *				Hiking	0.41	Forest T.	0.58	mean ± SD	mean ± SD
hematocrit	Time	9.23 (1.72, ∞)	<0.01 **		T1	0.53	Hi. x T1	0.46	FT. x T1	0.60	44.60 ± 2.21	45.62 ± 3.15
(g/dL)	Group*Time	1.18 (1.72, ∞)	0.30		T2	0.42	Hi. x T2	0.30	FT. x T2	0.53	43.10 ± 2.15	44.87 ± 2.53
	Group*T2	3.11 (1.00, ∞)	0.08	0.16	T3	0.54	Hi. x T3	0.47	FT. x T3	0.61	44.75 ± 2.34	45.78 ± 2.99
	Group*T3	0.02 (1.00, ∞)	0.90	0.90								
Reticulocytes	Group	0.07 (1.00, ∞)	0.79				Hiking	0.49	Forest T.	0.51	mean ± SD	mean ± SD
(%)	Time	24.78 (1.98, ∞)	<0.01 **		T1	0.46	Hi. x T1	0.47	FT. x T1	0.46	1.51 ± 0.38	1.50 ± 0.35
	Group*Time	0.65 (1.98, ∞)	0.52		T2	0.61	Hi. x T2	0.60	FT. x T2	0.61	1.71 ± 0.41	1.72 ± 0.48
	Group*T2	0.23 (1.00, ∞)	0.63	0.63	T3	0.43	Hi. x T3	0.41	FT. x T3	0.45	1.42 ± 0.38	1.49 ± 0.36
	Group*T3	0.18 (1.00, ∞)	0.28	0.56								
Immature	Group	3.37 (1.00, ∞)	0.07				Hiking	0.54	Forest T.	0.46	mean ± SD	mean ± SD
reticulocyte	Time	73.35 (1.98, ∞)	<0.01 **		T1	0.47	Hi. x T1	0.49	FT. x T1	0.44	10.74 ± 4.10	9.98 ± 3.69
fraction (IRF)	Group*Time	2.99 (1.98, ∞)	0.05		T2	0.67	Hi. x T2	0.74	FT. x T2	0.59	14.64 ± 4.42	12.31 ± 4.91
(%)	Group*T2	4.35 (1.00, ∞)	0.04 *	0.07	T3	0.44	Hi. x T3	0.39	FT. x T3	0.35	9.06 ± 3.00	8.81 ± 3.41
	Group*T3	0.95 (1.00, ∞)	0.95	0.95								
Leukocytes	Group	0.21 (1.00, ∞)	0.65				Hiking	0.51	Forest T.	0.49	mean ± SD	mean ± SD
(10³ µL)	Time	14.76 (1.94, ∞)	<0.01 *		T1	0.53	Hi. x T1	0.54	FT. x T1	0.53	6.96 ± 1.47	7.10 ± 1.81
	Group*Time	0.47 (1.94, ∞)	0.62		T2	0.43	Hi. x T2	0.44	FT. x T2	0.41	6.43 ± 1.39	6.54 ± 1.93
	Group*T2	0.22 (1.00, ∞)	0.64	0.64	T3	0.54	Hi. x T3	0.56	FT. x T3	0.52	7.17 ± 1.76	6.89 ± 1.57
	Group*T3	1.27 (1.00, ∞)	0.26	0.52								
Female	Group	0.41 (1.00, ∞)	0.52				Hiking	0.53	Forest T.	0.47	mean ± SD	mean ± SD
aerobic	Time	4.94 (1.94, ∞)	0.01 *		T1	0.46	Hi. x T1	0.42	FT. x T1	0.49	29.71 ± 3.73	30.31 ± 5.60
capacity	Group*Time	4.68 (1.94, ∞)	0.01 *		T2	0.59	Hi. x T2	0.70	FT. x T2	0.48	33.56 ± 4.18	30.19 ± 5.29
(mlO_2_/kg/min)	Group*T2	7.95 (1.00, ∞)	<0.01 **	0.01 *	T3	0.46	Hi. x T3	0.53	FT. x T3	0.45	30.90 ± 3.87	29.60 ± 5.96
	Group*T3	1.11 (1.00, ∞)	0.29	0.29								
Male	Group	0.87 (1.00, ∞)	0.35				Hiking	0.46	Forest T.	0.54	mean ± SD	mean ± SD
aerobic	Time	3.49 (1.72, ∞)	0.04 *		T1	0.42	Hi. x T1	0.43	FT. x T1	0.42	31.07 ± 4.73	31.13 ± 6.04
capacity	Group*Time	0.88 (1.72, ∞)	0.40		T2	0.58	Hi. x T2	0.52	FT. x T2	0.65	33.21 ± 6.17	34.86 ± 4.85
(mlO_2_/kg/min)	Group*T2	0.96 (1.00, ∞)	0.33	0.38	T3	0.48	Hi. x T3	0.42	FT. x T3	0.54	30.90 ± 3.87	32.39 ± 6.97
	Group*T3	1.72 (1.00, ∞)	0.19	0.38								

F1-LD-F1 model with time and treatment (forest therapy or hiking) and the interaction of treatment and time (treat_ time); Measuring times: T1 = day 0 baseline measurement, T2 = day 7 after intervention, T3 = day 60 follow-up, T4 = day 180 follow-up; ** <0.01, * <0.05, n. s./not significant ≥0.05; Abbreviations: adj. *p*: Holm-Bonferroni corrected *p*-value, F: F-Value, FT.: forest therapy group, Hi.: hiking group, *p*: *p*-value, RTE: Relative Treatment Effects, SD: standard deviation.

## Data Availability

The data presented in this study are available on request from the corresponding author.

## Supplementary Materials

The following supporting information can be downloaded at: https://www.mdpi.com/article/10.3390/ijerph20021469/s1, Table S1. Baseline characteristics of the study population regarding body parameters; Table S2. Baseline characteristics of the study population regarding questionnaires; Table S3. Results from the F1-LD-F1-model for balance, vital parameters and skin quality; Table S4. Results from the F1-LD-F1-model for body composition; Table S5. Results from the F1-LD-F1-model for questionnaires.

## References

[1] Warburton D.E.R., Bredin S.S.D. (2017). Health Benefits of Physical Activity: A Systematic Review of Current Systematic Reviews. Curr. Opin. Cardiol..

[2] Arem H., Moore S.C., Patel A., Hartge P., de Gonzalez A.B., Visvanathan K., Campbell P.T., Freedman M., Weiderpass E., Adami H.O. (2015). Leisure Time Physical Activity and Mortality: A Detailed Pooled Analysis of the Dose-Response Relationship. JAMA Intern. Med..

[3] Peluso M.A.M., Andrade L.H.S.G. (2005). de Physical Activity and Mental Health: The Association between Exercise and Mood. Clinics.

[4] Ruegsegger G.N., Booth F.W. (2018). Health Benefits of Exercise. Cold Spring Harb. Perspect. Med..

[5] Sato M., Du J., Inoue Y., Funk D.C., Weaver F. (2020). Older Adults’ Physical Activity and Healthcare Costs, 2003–2014. Am. J. Prev..

[6] (2012). Sedentary Behaviour Research Network Letter to the Editor: Standardized Use of the Terms “Sedentary” and “Sedentary Behaviours”. Appl. Physiol. Nutr. Metab..

[7] Ildefonzo Arocha Rodulfo J. (2019). Sedentarismo, la enfermedad del siglo xxi. Clín. Investig. Arterioscler..

[8] Martins L.C.G., de Lopes M.V.O., Diniz C.M., Guedes N.G. (2021). The Factors Related to a Sedentary Lifestyle: A Meta-analysis Review. J. Adv. Nurs..

[9] Després J.-P. (2016). Physical Activity, Sedentary Behaviours, and Cardiovascular Health: When Will Cardiorespiratory Fitness Become a Vital Sign?. Can. J. Cardiol..

[10] Blüher M. (2019). Obesity: Global Epidemiology and Pathogenesis. Nat. Rev. Endocrinol..

[11] World Health Organization (2022). Regional Office for Europe Supplement to the European Health Report 2021: Projections for a Selection of Indicators for Health-Related Sustainable Development Goals.

[12] Guthold R., Stevens G.A., Riley L.M., Bull F.C. (2018). Worldwide Trends in Insufficient Physical Activity from 2001 to 2016: A Pooled Analysis of 358 Population-Based Surveys with 1·9 Million Participants. Lancet Glob. Health.

[13] Granger E., Di Nardo F., Harrison A., Patterson L., Holmes R., Verma A. (2017). A Systematic Review of the Relationship of Physical Activity and Health Status in Adolescents. Eur. J. Public Health.

[14] Martínez-de-Quel Ó., Suárez-Iglesias D., López-Flores M., Pérez C.A. (2021). Physical Activity, Dietary Habits and Sleep Quality before and during COVID-19 Lockdown: A Longitudinal Study. Appetite.

[15] Fukushima N., Machida M., Kikuchi H., Amagasa S., Hayashi T., Odagiri Y., Takamiya T., Inoue S. (2021). Associations of Working from Home with Occupational Physical Activity and Sedentary Behavior under the COVID-19 Pandemic. J. Occup. Health.

[16] Holt-Lunstad J. (2018). Why Social Relationships Are Important for Physical Health: A Systems Approach to Understanding and Modifying Risk and Protection. Annu. Rev. Psychol..

[17] Langgartner D., Lowry C.A., Reber S.O. (2019). Old Friends, Immunoregulation, and Stress Resilience. Pflugers Arch..

[18] Freidl J., Huber D., Braunschmid H., Romodow C., Pichler C., Weisböck-Erdheim R., Mayr M., Hartl A. (2020). Winter Exercise and Speleotherapy for Allergy and Asthma: A Randomized Controlled Clinical Trial. JCM.

[19] Hanski I., von Hertzen L., Fyhrquist N., Koskinen K., Torppa K., Laatikainen T., Karisola P., Auvinen P., Paulin L., Mäkelä M.J. (2012). Environmental Biodiversity, Human Microbiota, and Allergy Are Interrelated. Proc. Natl. Acad. Sci. USA.

[20] Blaser M.J. (2017). The Theory of Disappearing Microbiota and the Epidemics of Chronic Diseases. Nat. Rev. Immunol..

[21] Rook G.A.W., Lowry C.A., Raison C.L. (2013). Microbial ‘Old Friends’, Immunoregulation and Stress Resilience. Evol. Med. Public Health.

[22] Mcdade T.W., Tallman P.S., Madimenos F.C., Liebert M.A., Cepon T.J., Sugiyama L.S., Snodgrass J.J. (2012). Analysis of Variability of High Sensitivity C-reactive Protein in Lowland Ecuador Reveals No Evidence of Chronic Low-grade Inflammation. Am. J. Hum. Biol..

[23] Lowry C.A., Smith D.G., Siebler P.H., Schmidt D., Stamper C.E., Hassell J.E., Yamashita P.S., Fox J.H., Reber S.O., Brenner L.A. (2016). The Microbiota, Immunoregulation, and Mental Health: Implications for Public Health. Curr. Envir. Health Rep..

[24] United Nations, Department of Economic and Social Affairs, Population Division (2019). World Urbanization Prospects: The 2018 Revision.

[25] Faulhaber M., Pocecco E., Niedermeier M., Ruedl G., Walter D., Sterr R., Ebner H., Schobersberger W., Burtscher M. (2017). Fall-Related Accidents among Hikers in the Austrian Alps: A 9-Year Retrospective Study. BMJ Open Sport Exerc. Med..

[26] Burtscher M. (2004). Endurance Performance of the Elderly Mountaineer: Requirements, Limitations, Testing, and Training. Wien. Klin. Wochenschr..

[27] Richins H., Hull J.S. (2016). Mountain Tourism: Experiences, Communities, Environments and Sustainable Futures.

[28] Evju M., Hagen D., Jokerud M., Olsen S.L., Selvaag S.K., Vistad O.I. (2021). Effects of Mountain Biking versus Hiking on Trails under Different Environmental Conditions. J. Environ. Manag..

[29] Pomfret G. (2006). Mountaineering Adventure Tourists: A Conceptual Framework for Research. Tour. Manag..

[30] Niedermeier M., Grafetstätter C., Kopp M., Huber D., Mayr M., Pichler C., Hartl A. (2019). The Role of Anthropogenic Elements in the Environment for Affective States and Cortisol Concentration in Mountain Hiking—A Crossover Trial. Int. J. Environ. Res. Public Health.

[31] Schobersberger W., Leichtfried V., Mueck-Weymann M., Humpeler E. (2010). Austrian Moderate Altitude Studies (AMAS): Benefits of Exposure to Moderate Altitudes (1500–2500 m). Sleep Breath.

[32] Yeon P.-S., Jeon J.-Y., Jung M.-S., Min G.-M., Kim G.-Y., Han K.-M., Shin M.-J., Jo S.-H., Kim J.-G., Shin W.-S. (2021). Effect of Forest Therapy on Depression and Anxiety: A Systematic Review and Meta-Analysis. Int. J. Environ. Res. Public Health.

[33] Oh K.H., Shin W.S., Khil T.G., Kim D.J. (2020). Six-Step Model of Nature-Based Therapy Process. Int. J. Environ. Res. Public Health.

[34] Chae Y., Lee S., Jo Y., Kang S., Park S., Kang H. (2021). The Effects of Forest Therapy on Immune Function. Int. J. Environ. Res. Public Health.

[35] Roviello V., Roviello G.N. (2022). Less COVID-19 Deaths in Southern and Insular Italy Explained by Forest Bathing, Mediterranean Environment, and Antiviral Plant Volatile Organic Compounds. Environ. Chem. Lett..

[36] Stier-Jarmer M., Throner V., Kirschneck M., Immich G., Frisch D., Schuh A. (2021). The Psychological and Physical Effects of Forests on Human Health: A Systematic Review of Systematic Reviews and Meta-Analyses. Int. J. Environ. Res. Public Health.

[37] Hansen M.M., Jones R., Tocchini K. (2017). Shinrin-Yoku (Forest Bathing) and Nature Therapy: A State-of-the-Art Review. Int. J. Environ. Res. Public Health.

[38] Wen Y., Yan Q., Pan Y., Gu X., Liu Y. (2019). Medical Empirical Research on Forest Bathing (Shinrin-Yoku): A Systematic Review. Environ. Health Prev. Med..

[39] Oh B., Lee K.J., Zaslawski C., Yeung A., Rosenthal D., Larkey L., Back M. (2017). Health and Well-Being Benefits of Spending Time in Forests: Systematic Review. Environ. Health Prev. Med..

[40] Pichler C., Freidl J., Bischof M., Kiem M., Erdheim-Weißböck R., Huber D., Squarra G., Murschetz P., Hartl A. (2022). Mountain Hiking vs. Forest Therapy. A Study Protocol of Novel Types of Nature-Based Intervention. Int. J. Environ. Res. Public Health.

[41] Vectorstall Man Free Icon. https://www.flaticon.com.

[42] Nadiinko Sleeping Icon. https://www.flaticon.com.

[43] Noguchi K., Gel Y.R., Brunner E., Konietschke F. (2012). NparLD: An R Software Package for the Nonparametric Analysis of Longitudinal Data in Factorial Experiments. J. Stat. Soft..

[44] Kraus D. (2014). Consolidated Data Analysis and Presentation Using an Open-Source Add-in for the Microsoft Excel^®^ Spreadsheet Software. Medical Writing.

[45] Prossegger J. (2019). Effects of Moderate Mountain Hiking and Balneotherapy on Community-Dwelling Older People: A Randomized Controlled Trial. Exp. Gerontol..

[46] Morse J.W., Gladkikh T.M., Hackenburg D.M., Gould R.K. (2020). COVID-19 and Human-Nature Relationships: Vermonters’ Activities in Nature and Associated Nonmaterial Values during the Pandemic. PLoS ONE.

[47] Almeida M., Shrestha A.D., Stojanac D., Miller L.J. (2020). The Impact of the COVID-19 Pandemic on Women’s Mental Health. Arch. Women’s Ment. Health.

[48] Smith D.T., Mouzon D.M., Elliott M. (2018). Reviewing the Assumptions About Men’s Mental Health: An Exploration of the Gender Binary. Am. J. Men’s Health.

[49] Estlein R., Gewirtz-Meydan A., Opuda E. (2022). Love in the Time of COVID-19: A Systematic Mapping Review of Empirical Research on Romantic Relationships One Year into the COVID-19 Pandemic. Fam. Process.

[50] Mallet R.T., Burtscher J., Richalet J.-P., Millet G.P., Burtscher M. (2021). Impact of High Altitude on Cardiovascular Health: Current Perspectives. Vasc. Health Risk Manag..

[51] Gatterer H., Raab C., Pramsohler S., Faulhaber M., Burtscher M., Netzer N. (2015). Effect of Weekly Hiking on Cardiovascular Risk Factors in the Elderly. Z. Für Gerontol. Und Geriatr..

[52] Neumayr G., Fries D., Mittermayer M., Humpeler E., Klingler A., Schobersberger W., Spiesberger R., Pokan R., Schmid P., Berent R. (2014). Effects of Hiking at Moderate and Low Altitude on Cardiovascular Parameters in Male Patients With Metabolic Syndrome: Austrian Moderate Altitude Study. Wilderness Environ. Med..

[53] Stoltzfus K.B., Naylor D., Cattermole T., Ankeney A., Mount R., Chang R., Gibson C.A. (2020). Blood Pressure Changes While Hiking at Moderate Altitudes: A Prospective Cohort Study. Int. J. Environ. Res. Public Health.

[54] Kim J.-G., Shin W.-S. (2021). Forest Therapy Alone or with a Guide: Is There a Difference between Self-Guided Forest Therapy and Guided Forest Therapy Programs?. Int. J. Environ. Res. Public Health.

[55] Banerjee D., Rai M. (2020). Social Isolation in Covid-19: The Impact of Loneliness. Int. J. Soc. Psychiatry.

[56] Smith B., Lim M. (2020). How the COVID-19 Pandemic Is Focusing Attention on Loneliness and Social Isolation. Public Health Res. Pract..

[57] Castañeda-Babarro A., Arbillaga-Etxarri A., Gutiérrez-Santamaría B., Coca A. (2020). Physical Activity Change during COVID-19 Confinement. Int. J. Environ. Res. Public Health.

[58] Ya’qoub L., Elgendy I.Y., Pepine C.J. (2021). Sex and Gender Differences in COVID-19: More to Be Learned!. Am. Heart J. Plus.

[59] Amgalan A., Malinowski A.K., Othman M. (2021). COVID-19 and Sex/Gender-Specific Differences: Understanding the Discrimination. Semin. Thromb. Hemost..

[60] Koprowicz A., Korzeniewicz R., Pusz W., Baranowska M. (2022). Sociodemographic Determinants of Poles’ Attitudes towards the Forest during the COVID-19 Pandemic. Int. J. Environ. Res. Public Health.

[61] Park B.-J., Shin C.-S., Shin W.-S., Chung C.-Y., Lee S.-H., Kim D.-J., Kim Y.-H., Park C.-E. (2020). Effects of Forest Therapy on Health Promotion among Middle-Aged Women: Focusing on Physiological Indicators. Int. J. Environ. Res. Public Health.

[62] Kim H., Kim J., Ju H.J., Jang B.J., Wang T.K., Kim Y.I. (2020). Effect of Forest Therapy for Menopausal Women with Insomnia. Int. J. Environ. Res. Public Health.

[63] Song C., Ikei H., Kagawa T., Miyazaki Y. (2019). Effects of Walking in a Forest on Young Women. Int. J. Environ. Res. Public Health.

[64] Grilli G., Sacchelli S. (2020). Health Benefits Derived from Forest: A Review. Int. J. Environ. Res. Public Health.

[65] Chan A.-W., Tetzlaff J.M., Altman D.G., Laupacis A., Gøtzsche P.C., Krleža-Jerić K., Hróbjartsson A., Mann H., Dickersin K., Berlin J.A. (2013). SPIRIT 2013 Statement: Defining Standard Protocol Items for Clinical Trials. Ann. Intern. Med..

[66] Buckley R.C., Brough P. (2017). Nature, Eco, and Adventure Therapies for Mental Health and Chronic Disease. Front. Public Health.

[67] Buckley R.C., Brough P., Westaway D. (2018). Bringing Outdoor Therapies Into Mainstream Mental Health. Front. Public Health.

[68] Cuschieri S. (2019). The CONSORT Statement. Saudi J. Anaesth..

