# Supplementary material for: Long-Term Effects of Mountain Hiking vs. Forest Therapy on Physical and Mental Health of Couples: A Randomized Controlled Trial
Source: Int J Environ Res Public Health. 2023 Jan 13;20(2):1469. doi: 10.3390/ijerph20021469 (PMC9859399; doi:10.3390/ijerph20021469)
Supplement: Supplementary file 1 [file ijerph-20-01469-s001.zip › ijerph-2058178-supplementary.pdf]

Table S1. Baseline characteristics of the study population regarding body parameters

	Hiking Group (n = 42)		Forest Therapy Group (n = 46)		Baseline Tests	
	mean ± SD	median ± IQR	mean ± SD	median ± IQR		
Size (cm)	171.6 ± 9.44	171.45 ± 14.75	170.96 ± 9.81	171.5 ± 17.33	0.74	U-Test
Weight (kg)	83.67 ± 11.56	83.25 ± 15.98	85.22 ± 15.2	83.25 ± 19.4	0.59	T-Test
Hip circumference (cm)	101.34 ± 5.97	100.9 ± 8.43	103.27 ± 8.56	102.5 ± 11.98	0.22	T-Test
Waist circumference (cm)	97.86 ± 7.32	96.45 ± 11.4	101.65 ±12.65	102.45 ± 18.28	0.09	T-Test
HR (bpm)	67.29 ± 9.56	67.5 ± 10.5	70.22 ±11.73	68 ± 12.75	0.42	U-Test
HRmax (bpm)	161.33 ± 4.66	161 ± 6.25	160.61 ± 5.19	160 ± 5.75	0.6	T-Test
RRdia (mmHg)	83.38 ± 10.2	83 ± 15.5	82.83 ± 10.66	80 ± 14	0.8	T-Test
RRsys (mmHg)	135.40 ± 19.07	132 ± 15.75	132.3 ± 16.8	129.5 ± 25.75	0.52	U-Test
Blood glucose (mg/dl)	110.5 ± 18.64	107 ± 19.5	110 ± 27.01	104 ± 19.75	0.41	U-Test
SpO_2 (%)	97.24 ± 0.98	98 ± 1	97.48 ± 0.78	98 ± 1	0.32	U-Test
FeNO (ppb)	20.1 ± 11.58	18 ± 13.5	19.85 ± 10.45	17.5 ± 13.75	0.99	U-Test
VO_2max (ml/min)	30.39 ± 4.22	28.76 ± 5.58	30.72 ± 5.73	30.10 ± 9.1	0.95	U-Test
Resistance (Ω)	477.55 ± 60.18	456 ± 100.25	474 ± 66.7	470 ± 114.5	0.82	U-Test
Reactance (Ω)	49.19 ± 6.04	49 ± 8.5	50.54 ± 7.67	50 ± 9.75	0.36	T-Test
Skin hydration (rcu)	35.37 ± 24.81	30.5 ± 32.73	32.85 ± 19.48	34.7 ± 33.38	0.78	U-Test
TEPWL (g/m²/h)	48.73 ±31.68	45.2 ± 44.06	48.7 ± 33.56	42.48 ± 46	0.92	U-Test

*FeNO: Fractional exhaled Nitric Oxide, HR: Heart rate. HRmax: Heart rate maximum, rcu: Relative corneometer units; RRdia: Diastolic blood pressure, RRsys: Systolic blood pressure, SpO_2: Oxygen saturation of arterial blood, TEPWL: Transepithelial Water Loss, VO_2max: Maximal Oxygen Consumption; * $p < 0.05$.*

Table S2. Baseline characteristics of the study population regarding questionnaires

	Hiking Group (n = 42)		Forest Therapy Group (n = 46)		Baseline Tests	
	mean ± SD	median ± IQR	mean ± SD	median ± IQR		
BFI Extraversion	3.6 ± 0.94	3.5 ± 1	3.41 ± 0.9	3.5 ± 1.38	0.4	U-Test
BFI Conscientiousness	3.93 ± 0.79	4 ± 1	3.969 ± 0.75	4 ± 1	0.9	U-Test
BFI Neuroticism	2.48 ± 0.8	2.25 ± 1	2.84 ± 0.94	3 ± 1.5	0.08	U-Test
BFI Openness to experience	3.68 ± 0.85	3.5 ± 1.5	3.63 ± 1.02	3.75 ± 1.5	0.96	U-Test
BFI Agreeableness	3.54 ± 0.74	3.5 ± 1	3.53 ± 0.8	3.5 ± 0.88	0.76	U-Test
CNS	3.9 ± 0.56	3.96 ± 0.83	3.88 ± 0.61	3.85 ± 0.72	0.9	T-Test
EQ-5D-5L Index	0.9 ± 0.14	0.91 ± 0.09	0.9 ± 0.07	0.91 ± 0.02	0.16	U-Test
EQ-5D-5L VAS	82.14 ± 11.59	80 ± 10	80.87 ± 10.92	80 ± 20	0.47	U-Test
FEGK external	2.65 ± 0.58	2.6 ± 0.8	2.84 ± 0.84	2.8 ± 1.35	0.21	T-Test
FEGK internal	5 ± 0.49	5 ± 0.55	4.78 ±0.52	4.8 ± 0.8	0.04*	T-Test
FS-D	48.17 ± 4.71	47.5 ± 6.75	46.78 ± 4.64	47 ± 6	0.17	T-Test
INS	4.67 ± 1.28	5 ± 1.75	4.67 ± 1.49	4.5 ± 2	0.94	U-Test
MAAS Total	4.54 ± 0.61	4.6 ± 0.5	4.45 ± 0.67	4.43 ± 0.95	0.29	U-Test
NRS-6 Experience	3.93 ± 0.45	4 ± 0.67	3.85 ± 0.54	3.83 ± 0.83	0.49	T-Test
NRS-6 Perspective	3.92 ± 0.61	4 ± 0.86	3.91 ± 0.73	3.93 ± 1.29	1	U-Test
NRS-6 Self	4.22 ±0.5	4.31 ± 0.63	4.04 ± 0.58	4 ± 0.75	0.12	U-Test
NRS-6 Total	4.04 ± 0.4	4.1 ± 0.64	3.94 ± 0.48	3.9 ± 0.65	0.38	U-Test
PFB Togetherness/Communication	20.43 ± 4.97	21 ± 5.5	18.72 ± 5.51	19 ± 7.5	0.13	T-Test
PFB Happiness	3.93 ± 0.95	4 ± 2	4.14 ± 0.87	4 ± 1	0.31	U-Test
PFB Conflict behaviour	5.26 ± 3.34	4.5 ± 5	6.37 ± 5.38	5 ± 7	0.64	U-Test
PFB Tenderness	15.93 ± 6.89	17.5 ± 10.75	17.29 ± 5.26	16.5 ± 7	0.30	T-Test
PFB Total	61.1 ± 12.24	61.5 ± 20.5	59.64 ± 13.13	60.25 ± 18.5	0.59	T-Test
PL	0.81 ± 1.45	0 ± 1	1.043 ± 2.01	0 ± 1	0.72	U-Test
SF12 Mental Component	80.69 ± 11.29	81.48 ± 18.52	81.08 ±10.37	85.19 ± 11.11	0.79	U-Test
SF12 Physical Component	85.89 ± 8.8	87.5 ± 11.88	81.30 ± 9.51	85 ± 15	0.02*	U-Test
SF12 Total	82.90 ± 9.32	82.98 ± 14.36	81.17 ± 8.05	82.98 ± 10.64	0.35	U-Test
SWSL	28.17 ± 4.47	29 ± 5	28.89 ± 3.21	29 ± 4.75	0.76	U-Test

BFI-10: 10 Item Big Five Inventory, CNS: Connectedness to Nature Scale, EQ-5D-5L: Euro Quality of Life Questionnaire (Index variable and Visual analogue scale), FEGK: Questionnaire for the Collection of Health-Related Control Beliefs, FS-D: German Version of the Flourishing Scale, INS: Inclusion to Nature Scale, MAAS: Mindful Attention and Awareness Scale, NRS-6: Nature Relatedness Scale, PFB: Partnership Questionnaire, PL: Problem List, SF-12: Short Form Health Survey, SWLS: Satisfaction with Life Scale; $p < 0.05$.*

Table 3. Results from the F1-LD-F1-model for primary outcomes

Parameter	F1-LD-F1 Model				Relative Treatment Effects (RTE)						Descriptive Statistics					
		F	p	adj. p	Time		Hiking		Forest Therapy		Hiking			Forest Therapy		
SF-12	Group	2.89 (1.00, ∞)	0.09				Hiking	0.55	Forest T.	0.46	mean		SD	mean		SD
Total Score	Time	8.07 (2.61, ∞)	< 0.01**		T1	0.43	Hi. x T1	0.47	FT. x T1	0.40	82.90	±	9.32	81.17	±	8.05
	Group * Time	0.45 (2.61, ∞)	0.69		T2	0.53	Hi. x T2	0.57	FT. x T2	0.49	85.31	±	10.54	83.95	±	8.51
	Group * T2	0.04 (1.00, ∞)	0.84	0.84	T3	0.54	Hi. x T3	0.60	FT. x T3	0.48	86.88	±	8.07	83.49	±	8.33
	Group * T3	1.07 (1.00, ∞)	0.30	0.60	T4	0.51	Hi. x T4	0.56	FT. x T4	0.46	85.56	±	9.46	82.24	±	9.91
	Group * T4	0.43 (1.00, ∞)	0.51	0.60												
SF-12	Group	8.22 (1.00, ∞)	< 0.01**				Hiking	0.57	Forest T.	0.43	mean		SD	mean		SD
Physical	Time	12.48 (2.76, ∞)	< 0 01**		T1	0.41	Hi. x T1	0.48	FT. x T1	0.34	85.89	±	8.80	81.30	±	9.51
Component	Group * Time	0.78 (2.76, ∞)	0.05		T2	0.52	Hi. x T2	0.58	FT. x T2	0.46	88.57	±	9.13	85.22	±	9.31
	Group * T2	0.36 (1.00, ∞)	0.55	1.00	T3	0.55	Hi. x T3	0.61	FT. x T3	0.48	89.52	±	8.47	85.22	±	10.70
	Group * T3	0.02 (1.00, ∞)	0.89	1.00	T4	0.54	Hi. x T4	0.63	FT. x T4	0.44	90.36	±	7.52	84.13	±	10.66
	Group * T4	0.91 (1.00, ∞)	0.34	1.00												
SF-12	Group	0.39 (1.00, ∞)	0.53				Hiking	0.52	Forest T.	0.48	mean		SD	mean		SD
Mental	Time	3.31 (2.66, ∞)	0.02*		T1	0.46	Hi. x T1	0.46	FT. x T1	0.46	80.69	±	11.29	81.08	±	10.37
Component	Group * Time	0.69 (2.66, ∞)	0.54		T2	0.53	Hi. x T2	0.54	FT. x T2	0.51	82.89	±	13.34	83.01	±	10.52
	Group * T2	0.73 (1.00, ∞)	0.39	0.78	T3	0.53	Hi. x T3	0.57	FT. x T3	0.49	84.92	±	9.62	82.21	±	10.14
	Group * T3	2.24 (1.00, ∞)	0.13	0.40	T4	0.49	Hi. x T4	0.51	FT. x T4	0.48	82.01	±	12.56	80.84	±	12.18
	Group * T4	0.32 (1.00, ∞)	0.40	0.57												
EQ5D-5L	Group	0.40 (1.00, ∞)	0.53				Hiking	0.52	Forest T.	0.49	mean		SD	mean		SD
Visual	Time	11.25 (2.80, ∞)	< 0.01**		T1	0.43	Hi. x T1	0.45	FT. x T1	0.40	82.14	±	11.59	80.87	±	10.92
Analogue	Group * Time	0.15 (2.80, ∞)	0.92		T2	0.58	Hi. x T2	0.58	FT. x T2	0.57	87.38	±	10.83	85.22	±	15.74
Scale (VAS)	Group * T2	0.33 (1.00, ∞)	0.57	1.00	T3	0.52	Hi. x T3	0.54	FT. x T3	0.51	85.95	±	9.64	83.26	±	13.34
	Group * T3	0.02 (1.00, ∞)	0.90	1.00	T4	0.48	Hi. x T4	0.50	FT. x T4	0.46	84.05	±	12.31	81.74	±	12.88
	Group * T4	0.00 (1.00, ∞)	0.99	1.00												
EQ5D-5L	Group	5.20 (1.00, ∞)	0.02*				Hiking	0.56	Forest T.	0.45	mean		SD	mean		SD
Index	Time	10.19 (2.78, ∞)	< 0.01**		T1	0.44	Hi. x T1	0.48	FT. x T1	0.39	0.90	±	0.14	0.90	±	0.07
	Group * Time	0.88 (2.78, ∞)	0.45		T2	0.47	Hi. x T2	0.52	FT. x T2	0.42	0.93	±	0.07	0.90	±	0.08
	Group * T2	0.01 (1.00, ∞)	0.92	1.00	T3	0.57	Hi. x T3	0.62	FT. x T3	0.52	0.94	±	0.07	0.93	±	0.06
	Group * T3	0.06 (1.00, ∞)	0.81	1.00	T4	0.53	Hi. x T4	0.61	FT. x T4	0.45	0.95	±	0.06	0.90	±	0.09
	Group * T4	1.80 (1.00, ∞)	0.18	1.00												
Partnership	Group	0.26 (1.00, ∞)	0.61				Hiking	0.52	Forest T.	0.49	mean		SD	mean		SD
Questionnaire	Time	5.71 (2.65, ∞)	< 0.01**		T1	0.50	Hi. x T1	0.51	FT. x T1	0.48	61.10	±	12.24	59.64	±	13.13
Total Score	Group * Time	0.15 (2.65, ∞)	0.91		T2	0.53	Hi. x T2	0.54	FT. x T2	0.51	62.64	±	14.06	60.62	±	13.62
	Group * T2	0.00 (1.00, ∞)	0.99	1.00	T3	0.52	Hi. x T3	0.53	FT. x T3	0.51	62.31	±	13.29	60.48	±	13.51
	Group * T3	0.01 (1.00, ∞)	0.91	1.00	T4	0.46	Hi. x T4	0.48	FT. x T4	0.44	60.02	±	15.16	57.02	±	14.91
	Group * T4	0.13 (1.00, ∞)	0.72	1.00												
Partnership	Group	0.52 (1.00, ∞)	0.47				Hiking	0.48	Forest T.	0.52	mean		SD	mean		SD
Questionnaire	Time	5.69 (2.55, ∞)	< 0.01**		T1	0.52	Hi. x T1	0.49	FT. x T1	0.55	3.93	±	0.95	4.14	±	0.86
Happiness	Group * Time	2.51 (2.55, ∞)	0.07		T2	0.52	Hi. x T2	0.51	FT. x T2	0.53	4.05	±	0.73	4.03	±	0.99
	Group * T2	1.17 (1.00, ∞)	0.28	0.84	T3	0.52	Hi. x T3	0.47	FT. x T3	0.57	3.81	±	1.11	4.20	±	0.83
	Group * T3	0.63 (1.00, ∞)	0.43	0.85	T4	0.44	Hi. x T4	0.45	FT. x T4	0.42	3.74	±	1.15	3.67	±	1.08
	Group * T4	2.33 (1.00, ∞)	0.13	0.85												
Problem List	Group	0.11 (1.00, ∞)	0.74				Hiking	0.49	Forest T.	0.51	mean		SD	mean		SD
	Time	3.43 (2.72, ∞)	0.02*		T1	0.52	Hi. x T1	0.51	FT. x T1	0.53	0.81	±	1.45	1.04	±	2.01
	Group * Time	3.12 (2.72, ∞)	0.03*		T2	0.49	Hi. x T2	0.47	FT. x T2	0.51	0.50	±	1.02	0.74	±	1.34
	Group * T2	0.30 (1.00, ∞)	0.58	1.00	T3	0.47	Hi. x T3	0.43	FT. x T3	0.50	0.38	±	1.08	0.91	±	2.29
	Group * T3	0.34 (1.00, ∞)	0.34	1.00	T4	0.53	Hi. x T4	0.56	FT. x T4	0.49	1.40	±	2.47	0.78	±	1.65
	Group * T4	3.09 (1.00, ∞)	0.08	1.00												

F1-LD-F1 model with group (Forest Therapy or hiking), time and the interaction of group and time (group*time); T1 = day 0/baseline, T2 = day 7/after intervention week, T3 = day 60/follow-up 1, T4 = day 180/follow-up 2; ** <0.01, * <0.05, n. s./not significant ≥0.05; Results for the Partnership Questionnaire Subscales dispute behaviour, tenderness and commonality/communication can be found in Table S5 in the supplemental materials. Abbreviations: adj. p: Holm-Bonferroni corrected p-value, EQ-5D-5L: Euro Quality of Life Questionnaire (Index variable and Visual analogue scale), F: F-Value, FT.: Forest Therapy group, Hi.: hiking group, p: p-value, RTE: Relative Treatment Effects, SD: standard deviation, SF-12: Short Form Health Survey;

Table 4. Results from the F1-LD-F1-model for differential blood count and aerobic capacity

Parameter		F1-LD-F1 Model			Relative Treatment Effects (RTE)						Descriptive Statistics					
		F	p	adj. p	Time		Hiking		Forest Therapy		Hiking			Forest Therapy		
Female	Group	0.26 (1.00, ∞)	0.61				Hiking	0.52	Forest T.	0.48	mean		SD	mean		SD
erythrocytes	Time	7.62 (1.83, ∞)	< 0.01**		T1	0.54	Hi. x T1	0.56	FT. x T1	0.52	4.67	±	0.36	4.61	±	0.27
(10^6µl)	Group * Time	7.37 (1.83, ∞)	< 0.01**		T2	0.44	Hi. x T2	0.40	FT. x T2	0.47	4.48	±	0.31	4.56	±	0.35
	Group * T2	5.45 (1.00, ∞)	0.02*	0.04*	T3	0.52	Hi. x T3	0.60	FT. x T3	0.45	4.74	±	0.41	4.52	±	0.28
	Group * T3	3.07 (1.00, ∞)	0.08	0.08												
Male	Group	1.85 (1.00, ∞)	0.17				Hiking	0.45	Forest T.	0.55	mean		SD	mean		SD
erythrocytes	Time	7.50 (1.74, ∞)	< 0.01**		T1	0.56	Hi. x T1	0.52	FT. x T1	0.60	4.88	±	0.29	4.99	±	0.40
(10^6µl)	Group * Time	1.01 (1.74, ∞)	0.36		T2	0.44	Hi. x T2	0.36	FT. x T2	0.52	4.70	±	0.28	4.87	±	0.33
	Group * T2	2.58 (1.00, ∞)	0.11	0.22	T3	0.49	Hi. x T3	0.45	FT. x T3	0.53	4.82	±	0.28	4.91	±	0.39
	Group * T3	0.01 (1.00, ∞)	0.92	0.92												
Female	Group	0.24 (1.00, ∞)	0.97				Hiking	0.50	Forest T.	0.50	mean		SD	mean		SD
haematocrit	Time	9.92 (1.89, ∞)	< 0.01**		T1	0.52	Hi. x T1	0.53	FT. x T1	0.51	42.01	±	2.73	41.87	±	2.08
(g/dl)	Group * Time	3.67 (1.89, ∞)	0.03*		T2	0.42	Hi. x T2	0.38	FT. x T2	0.47	40.57	±	2.80	41.46	±	2.68
	Group * T2	4.03 (1.00, ∞)	0.04*	0.09	T3	0.56	Hi. x T3	0.60	FT. x T3	0.52	42.74	±	3.05	41.83	±	2.19
	Group * T3	0.66 (1.00, ∞)	0.42	0.42												
Male	Group	5.23 (1.00, ∞)	0.02*				Hiking	0.41	Forest T.	0.58	mean		SD	mean		SD
haematocrit	Time	9.23 (1.72, ∞)	< 0.01**		T1	0.53	Hi. x T1	0.46	FT. x T1	0.60	44.60	±	2.21	45.62	±	3.15
(g/dl)	Group * Time	1.18 (1.72, ∞)	0.30		T2	0.42	Hi. x T2	0.30	FT. x T2	0.53	43.10	±	2.15	44.87	±	2.53
	Group * T2	3.11 (1.00, ∞)	0.08	0.16	T3	0.54	Hi. x T3	0.47	FT. x T3	0.61	44.75	±	2.34	45.78	±	2.99
	Group * T3	0.02 (1.00, ∞)	0.90	0.90												
Reticulocytes	Group	0.07 (1.00, ∞)	0.79				Hiking	0.49	Forest T.	0.51	mean		SD	mean		SD
(%)	Time	24.78 (1.98, ∞)	< 0.01**		T1	0.46	Hi. x T1	0.47	FT. x T1	0.46	1.51	±	0.38	1.50	±	0.35
	Group * Time	0.65 (1.98, ∞)	0.52		T2	0.61	Hi. x T2	0.60	FT. x T2	0.61	1.71	±	0.41	1.72	±	0.48
	Group * T2	0.23 (1.00, ∞)	0.63	0.63	T3	0.43	Hi. x T3	0.41	FT. x T3	0.45	1.42	±	0.38	1.49	±	0.36
	Group * T3	0.18 (1.00, ∞)	0.28	0.56												
Immature	Group	3.37 (1.00, ∞)	0.07				Hiking	0.54	Forest T.	0.46	mean		SD	mean		SD
reticulocyte	Time	73.35 (1.98, ∞)	< 0.01**		T1	0.47	Hi. x T1	0.49	FT. x T1	0.44	10.74	±	4.10	9.98	±	3.69
fraction (IRF)	Group * Time	2.99 (1.98, ∞)	0.05		T2	0.67	Hi. x T2	0.74	FT. x T2	0.59	14.64	±	4.42	12.31	±	4.91
(%)	Group * T2	4.35 (1.00, ∞)	0.04*	0.07	T3	0.44	Hi. x T3	0.39	FT. x T3	0.35	9.06	±	3.00	8.81	±	3.41
	Group * T3	0.95 (1.00, ∞)	0.95	0.95												
Leukocytes	Group	0.21 (1.00, ∞)	0.65				Hiking	0.51	Forest T.	0.49	mean		SD	mean		SD
(10^3µl)	Time	14.76 (1.94, ∞)	< 0.01*		T1	0.53	Hi. x T1	0.54	FT. x T1	0.53	6.96	±	1.47	7.10	±	1.81
	Group * Time	0.47 (1.94, ∞)	0.62		T2	0.43	Hi. x T2	0.44	FT. x T2	0.41	6.43	±	1.39	6.54	±	1.93
	Group * T2	0.22 (1.00, ∞)	0.64	0.64	T3	0.54	Hi. x T3	0.56	FT. x T3	0.52	7.17	±	1.76	6.89	±	1.57
	Group * T3	1.27 (1.00, ∞)	0.26	0.52												
Female	Group	0.41 (1.00, ∞)	0.52				Hiking	0.53	Forest T.	0.47	mean		SD	mean		SD
aerobic	Time	4.94 (1.94, ∞)	0.01*		T1	0.46	Hi. x T1	0.42	FT. x T1	0.49	29.71	±	3.73	30.31	±	5.60
capacity	Group * Time	4.68 (1.94, ∞)	0.01*		T2	0.59	Hi. x T2	0.70	FT. x T2	0.48	33.56	±	4.18	30.19	±	5.29
(mlO_2/kg/min)	Group * T2	7.95 (1.00, ∞)	< 0.01**	0.01*	T3	0.46	Hi. x T3	0.53	FT. x T3	0.45	30.90	±	3.87	29.60	±	5.96
	Group * T3	1.11 (1.00, ∞)	0.29	0.29												
Male	Group	0.87 (1.00, ∞)	0.35				Hiking	0.46	Forest T.	0.54	mean		SD	mean		SD
aerobic	Time	3.49 (1.72, ∞)	0.04*		T1	0.42	Hi. x T1	0.43	FT. x T1	0.42	31.07	±	4.73	31.13	±	6.04
capacity	Group * Time	0.88 (1.72, ∞)	0.40		T2	0.58	Hi. x T2	0.52	FT. x T2	0.65	33.21	±	6.17	34.86	±	4.85
(mlO_2/kg/min)	Group * T2	0.96 (1.00, ∞)	0.33	0.38	T3	0.48	Hi. x T3	0.42	FT. x T3	0.54	30.90	±	3.87	32.39	±	6.97
	Group * T3	1.72 (1.00, ∞)	0.19	0.38												

F1-LD-F1 model with time and treatment (Forest Therapy or hiking) and the interaction of treatment and time (treat_ time); Measuring times: T1 = day 0 baseline measurement, T2 = day 7 after intervention, T3 = day 60 follow-up, T4 = day 180 follow-up; ** <0.01, * <0.05, n. s./not significant ≥0.05; Abbreviations: adj. p: Holm-Bonferroni corrected p-value, F: F-Value, FT.: Forest Therapy group, Hi.: hiking group, p: p-value, RTE: Relative Treatment Effects, SD: standard deviation;

Table S5. Results from the F1-LD-F1-model for questionnaires

Parameter		F1-LD-F1 Model			Relative Treatment Effects (RTE)						Descriptive Statistics					
		F	p	adj. p	Time		Hiking		Forest Therapy		Hiking			Forest Therapy		
CNS female	Group	0.35 (1.00, ∞)	0.55				Hiking	0.53	Forest T.	0.48	mean		SD	mean		SD
	Time	4.71 (2.76, ∞)	< 0.01**		T1	0.47	Hi. x T1	0.47	FT. x T1	0.46	4.04	±	0.61	4.06	±	0.56
	Group * Time	0.92 (2.76, ∞)	0.42		T2	0.56	Hi. x T2	0.58	FT. x T2	0.53	4.25	±	0.55	4.18	±	0.52
	Group * T2	0.70 (1.00, ∞)	0.40	0.40	T3	0.51	Hi. x T3	0.56	FT. x T3	0.47	4.22	±	0.59	4.07	±	0.50
	Group * T3	3.54 (1.00, ∞)	0.06	0.18	T4	0.47	Hi. x T4	0.49	FT. x T4	0.45	4.11	±	0.53	4.03	±	0.55
	Group * T4	0.37 (1.00, ∞)	0.18	0.54												
CNS male	Group	0.38 (1.00, ∞)	0.54				Hiking	0.53	Forest T.	0.48	mean		SD	mean		SD
	Time	3.71 (2.71, ∞)	0.01*		T1	0.47	Hi. x T1	0.48	FT. x T1	0.46	3.77	±	0.57	3.71	±	0.63
	Group * Time	1.35 (2.71, ∞)	0.26		T2	0.55	Hi. x T2	0.56	FT. x T2	0.53	3.93	±	0.70	3.87	±	0.62
	Group * T2	0.06 (1.00, ∞)	0.81	1.00	T3	0.52	Hi. x T3	0.54	FT. x T3	0.51	3.89	±	0.48	3.80	±	0.67
	Group * T3	0.03 (1.00, ∞)	0.87	1.00	T4	0.46	Hi. x T4	0.53	FT. x T4	0.40	3.89	±	0.59	3.61	±	0.68
	Group * T4	3.25 (1.00, ∞)	0.07	1.00												
INS	Group	0.30 (1.00, ∞)	0.58				Hiking	0.48	Forest T.	0.51	mean		SD	mean		SD
	Time	3.23 (2.33, ∞)	0.03*		T1	0.46	Hi. x T1	0.45	FT. x T1	0.46	4.67	±	1.28	4.67	±	1.49
	Group * Time	0.13 (2.33, ∞)	0.90		T2	0.52	Hi. x T2	0.51	FT. x T2	0.54	4.93	±	1.24	5.02	±	1.44
	Group * T2	0.28 (1.00, ∞)	0.60	1.00	T3	0.51	Hi. x T3	0.49	FT. x T3	0.52	4.88	±	1.31	4.93	±	1.55
	Group * T3	0.09 (1.00, ∞)	0.77	1.00	T4	0.51	Hi. x T4	0.49	FT. x T4	0.53	4.79	±	1.42	5.02	±	1.36
	Group * T4	0.42 (1.00, ∞)	0.52	1.00												
NRS-6 Total	Group	0.03 (1.00, ∞)	0.58				Hiking	0.51	Forest T.	0.50	mean		SD	mean		SD
	Time	1.31 (2.33, ∞)	0.03*		T1	0.48	Hi. x T1	0.51	FT. x T1	0.45	4.04	±	0.40	3.94	±	0.48
	Group * Time	1.52 (2.33, ∞)	0.90		T2	0.52	Hi. x T2	0.51	FT. x T2	0.53	4.03	±	0.42	4.04	±	0.49
					T3	0.49	Hi. x T3	0.47	FT. x T3	0.50	3.97	±	0.47	4.02	±	0.47
					T4	0.51	Hi. x T4	0.53	FT. x T4	0.50	4.05	±	0.45	4.02	±	0.46
MAAS Total	Group	1.80 (1.00, ∞)	0.18				Hiking	0.54	Forest T.	0.47	mean		SD	mean		SD
	Time	4.52 (2.77, ∞)	< 0.01**		T1	0.46	Hi. x T1	0.48	FT. x T1	0.44	4.54	±	0.61	4.45	±	0.67
	Group * Time	0.29 (2.77, ∞)	0.81		T2	0.49	Hi. x T2	0.53	FT. x T2	0.45	4.67	±	0.57	4.48	±	0.70
	Group * T2	0.38 (1.00, ∞)	0.54	1.00	T3	0.54	Hi. x T3	0.58	FT. x T3	0.50	4.75	±	0.62	4.59	±	0.61
	Group * T3	0.23 (1.00, ∞)	0.63	1.00	T4	0.52	Hi. x T4	0.56	FT. x T4	0.48	4.68	±	0.73	4.53	±	0.76
	Group * T4	0.48 (1.00, ∞)	0.49	1.00												
SWLS	Group	1.32 (1.00, ∞)	0.25				Hiking	0.53	Forest T.	0.47	mean		SD	mean		SD
	Time	3.15 (2.80, ∞)	0.03*		T1	0.50	Hi. x T1	0.49	FT. x T1	0.50	2.65	±	0.58	2.84	±	0.84
	Group * Time	3.11 (2.80, ∞)	0.03*		T2	0.54	Hi. x T2	0.56	FT. x T2	0.51	2.69	±	0.96	2.70	±	1.10
	Group * T2	2.13 (1.00, ∞)	0.14	0.29	T3	0.52	Hi. x T3	0.59	FT. x T3	0.44	2.88	±	0.81	2.77	±	0.92
	Group * T3	7.27 (1.00, ∞)	0.01*	0.02*	T4	0.46	Hi. x T4	0.48	FT. x T4	0.43	2.95	±	0.98	2.83	±	1.03
	Group * T4	1.67 (1.00, ∞)	0.02*	0.20												
FEGK Internal	Group	6.70 (1.00, ∞)	0.01*				Hiking	0.57	Forest T.	0.44	mean		SD	mean		SD
	Time	3.19 (2.85, ∞)	0.02*		T1	0.50	Hi. x T1	0.56	FT. x T1	0.44	5.00	±	0.49	4.78	±	0.52
	Group * Time	0.98 (2.85, ∞)	0.40		T2	0.55	Hi. x T2	0.61	FT. x T2	0.48	5.12	±	0.52	4.85	±	0.53
	Group * T2	0.20 (1.00, ∞)	0.65	0.65	T3	0.49	Hi. x T3	0.58	FT. x T3	0.40	5.07	±	0.54	4.70	±	0.49
	Group * T3	1.74 (1.00, ∞)	0.19	0.38	T4	0.47	Hi. x T4	0.53	FT. x T4	0.42	4.97	±	0.63	4.74	±	0.66
	Group * T4	0.00 (1.00, ∞)	0.38	0.98												
FEGK External	Group	0.00 (1.00, ∞)	0.98				Hiking	0.50	Forest T.	0.50	mean		SD	mean		SD
	Time	3.96 (2.75, ∞)	0.01*		T1	0.49	Hi. x T1	0.46	FT. x T1	0.52	2.65	±	0.58	2.84	±	0.84
	Group * Time	2.11 (2.75, ∞)	0.10		T2	0.46	Hi. x T2	0.46	FT. x T2	0.46	2.69	±	0.96	2.70	±	1.10
	Group * T2	1.96 (1.00, ∞)	0.16	0.32	T3	0.51	Hi. x T3	0.53	FT. x T3	0.50	2.88	±	0.81	2.77	±	0.92
	Group * T3	5.14 (1.00, ∞)	0.02*	0.07	T4	0.53	Hi. x T4	0.54	FT. x T4	0.52	2.95	±	0.98	2.83	±	1.03
	Group * T4	4.30 (1.00, ∞)	0.04*	0.07												
IPAQ-SF Total	Group	0.50 (1.00, ∞)	0.48				Hiking	0.48	Forest T.	0.52	mean		SD	mean		SD
	Time	4.27 (1.94, ∞)	0.01*		T1	0.44	Hi. x T1	0.40	FT. x T1	0.48	2598.2	±	2929.3	4917.3	±	7621.2
	Group * Time	0.88 (1.94, ∞)	0.41		T3	0.52	Hi. x T3	0.53	FT. x T3	0.51	3575.4	±	2746.2	4257.9	±	4598.6
	Group * T3	1.93 (1.00, ∞)	0.16	0.33	T4	0.53	Hi. x T4	0.51	FT. x T4	0.55	4724.5	±	8575.0	5041.9	±	5295.3
	Group * T4	0.31 (1.00, ∞)	0.33	0.58												
IPAQ-SF Cat.	Group	0.02 (1.00, ∞)	0.89				Hiking	0.50	Forest T.	0.50	mean		SD	mean		SD
	Time	5.36 (1.97, ∞)	< 0.01**		T1	0.44	Hi. x T1	0.42	FT. x T1	0.46	2.00	±	0.80	2.11	±	0.80
	Group * Time	0.39 (1.97, ∞)	0.67		T3	0.52	Hi. x T3	0.53	FT. x T3	0.51	2.31	±	0.78	2.26	±	0.77
	Group * T3	0.74 (1.00, ∞)	0.39	0.78	T4	0.54	Hi. x T4	0.54	FT. x T4	0.54	2.33	±	0.79	2.33	±	0.82
	Group * T4	0.30 (1.00, ∞)	0.58	0.78												
Female	Group	1.47 (1.00, ∞)	0.23				Hiking	0.55	Forest T.	0.46	mean		SD	mean		SD
Flourishing	Time	4.01 (2.66, ∞)	0.01*		T1	0.45	Hi. x T1	0.47	FT. x T1	0.42	48.67	±	4.75	47.70	±	4.32
Scale	Group * Time	0.52 (2.66, ∞)	0.65		T2	0.55	Hi. x T2	0.61	FT. x T2	0.49	50.29	±	3.91	48.52	±	3.93

	Group * T2	1.18 (1.00, ∞)	0.28	0.83	T3	0.53	Hi. x T3	0.58	FT. x T3	0.49	50.00	±	3.49	48.57	±	3.53
	Group * T3	0.16 (1.00, ∞)	0.69	1.00	T4	0.48	Hi. x T4	0.54	FT. x T4	0.42	49.19	±	4.41	47.39	±	4.62
	Group * T4	0.93 (1.00, ∞)	0.33	1.00												
Male Flourishing Scale	Group	2.45 (1.00, ∞)	0.12				Hiking	0.56	Forest T.	0.44	mean		SD	mean		SD
	Time	0.56 (2.86, ∞)	0.63		T1	0.51	Hi. x T1	0.54	FT. x T1	0.47	47.67	±	4.73	45.87	±	4.86
	Group * Time	1.23 (2.86, ∞)	0.30		T2	0.51	Hi. x T2	0.60	FT. x T2	0.42	48.43	±	4.80	45.43	±	5.08
	Group * T3	2.90 (1.00, ∞)	0.06	0.27	T3	0.52	Hi. x T3	0.59	FT. x T3	0.44	48.38	±	4.67	45.78	±	4.75
	Group * T4	1.27 (1.00, ∞)	0.26	0.52	T4	0.48	Hi. x T4	0.52	FT. x T4	0.43	47.33	±	4.94	45.61	±	4.86
		0.03 (1.00, ∞)	0.85	0.52												
NRS-6 Self Female	Group	0.01 (1.00, ∞)	0.92				Hiking	0.50	Forest T.	0.50	mean		SD	mean		SD
	Time	0.54 (2.77, ∞)	0.64		T1	0.48	Hi. x T1	0.51	FT. x T1	0.46	4.29	±	0.54	4.20	±	0.57
	Group * Time	0.83 (2.77, ∞)	0.47		T2	0.52	Hi. x T2	0.53	FT. x T2	0.51	4.32	±	0.51	4.29	±	0.48
					T3	0.48	Hi. x T3	0.49	FT. x T3	0.47	4.27	±	0.52	4.25	±	0.46
					T4	0.52	Hi. x T4	0.49	FT. x T4	0.55	4.28	±	0.46	4.38	±	0.45
NRS-6 Self Male	Group	2.35 (1.00, ∞)	0.13				Hiking	0.56	Forest T.	0.45	mean		SD	mean		SD
	Time	0.17 (2.78, ∞)	0.90		T1	0.49	Hi. x T1	0.56	FT. x T1	0.41	4.15	±	0.46	3.88	±	0.57
	Group * Time	0.69 (2.78, ∞)	0.55		T2	0.52	Hi. x T2	0.56	FT. x T2	0.47	4.15	±	0.53	3.96	±	0.67
					T3	0.50	Hi. x T3	0.53	FT. x T3	0.47	4.11	±	0.56	3.95	±	0.63
					T4	0.50	Hi. x T4	0.58	FT. x T4	0.43	4.18	±	0.57	3.91	±	0.53
NRS-6 Self Perspective	Group	0.46 (1.00, ∞)	0.50				Hiking	0.48	Forest T.	0.52	mean		SD	mean		SD
	Time	0.54 (2.91, ∞)	0.65		T1	0.49	Hi. x T1	0.48	FT. x T1	0.49	3.92	±	0.61	3.91	±	0.73
	Group * Time	0.73 (2.91, ∞)	0.53		T2	0.51	Hi. x T2	0.49	FT. x T2	0.54	3.91	±	0.62	4.02	±	0.70
					T3	0.50	Hi. x T3	0.46	FT. x T3	0.53	3.86	±	0.66	4.01	±	0.67
					T4	0.50	Hi. x T4	0.49	FT. x T4	0.51	3.91	±	0.64	3.99	±	0.61
NRS-6 Self Experience	Group	0.00 (1.00, ∞)	0.95				Hiking	0.50	Forest T.	0.50	mean		SD	mean		SD
	Time	0.73 (2.98, ∞)	0.53		T1	0.49	Hi. x T1	0.51	FT. x T1	0.48	3.93	±	0.45	3.85	±	0.54
	Group * Time	1.39 (2.98, ∞)	0.24		T2	0.51	Hi. x T2	0.50	FT. x T2	0.53	3.90	±	0.46	3.96	±	0.47
					T3	0.48	Hi. x T3	0.45	FT. x T3	0.51	3.80	±	0.54	3.93	±	0.53
					T4	0.52	Hi. x T4	0.54	FT. x T4	0.49	3.96	±	0.58	3.89	±	0.53

F1-LD-F1 model with time and treatment (Forest Therapy or hiking) and the interaction of treatment and time (treat_ time); Measuring times: T1 = day 0 baseline measurement, T2 = day 7 after intervention, T3 = day 60 follow-up, T4 = day 180 follow-up; ** <0.01, * <0.05, n. s./not significant ≥0.05; Abbreviations: adj. p: Holm-Bonferroni corrected p-value, CNS: Connectedness to Nature Scale, F: F-Value, FEGK: Questionnaire for the Collection of Health-Related Control internal and external Beliefs, FT.: Forest Therapy group, Hi.: hiking group, INS: Inclusion to Nature Scale, IPAQ-SF: Total and Category Score of International Physical Activity Questionnaire Short Form, MAAS: Mindful Attention and Awareness Scale, NRS-6: Score of Nature Relatedness Scale, p: p-value, RTE: Relative Treatment Effects, SD: standard deviation, SWLS: Satisfaction with Life Scale;
